# Supplementary material for: A glacial survivor of the alpine Mediterranean region: phylogenetic and phylogeographic insights into Silene ciliata Pourr. (Caryophyllaceae)
Source: PeerJ. 2015 Aug 20;3:e1193. doi: 10.7717/peerj.1193 (PMC4548490; doi:10.7717/peerj.1193)
Supplement: Data Set S1 — The alignments obtained after multiply aligning the cpDNA sequences of S. ciliata samples with ClustalW. These alignments were then used for the conduction of all the analyses. [file peerj-03-1193-s004.docx]

>Cen2 AAGTGTTGGATTTAAAGCTGGTGTTAAAGATTACAAATTGACTTATTATACTCCTGAGTATGAAACCCTAGATACTGATATCTTGGCAGCATTCCGAGTAACTCCACAACCCGGAGTTCCACCCGAAGAAGCAGGGGCCGCAGTAGCCGCCGAATCTTCTACGGGTACATGGACAACTGTATGGACCGACGGACTTACCAGTCTTGATCGTTACAAAGGACGATGCTACCACATCGAGCCTGTTGCTGGAGAGGAAAATCAATATATTTGTTATGTAGCTTACCCCTTAGACCTTTTTGAGGAAGGCTCTGTTACTAACATGTTTACTTCCATTGTGGGTAATGTATTTGGGTTCAAAGCCTTGCGTGCTCTACGTTTGGAGGATTTGCGAATCCCTGTTGCTTATGTAAAAACTTTCCTAGGCCCGCCTCACGGTATCCAAGTTGAAAGAGATAAATTGAACAAGTATGGCCGTCCCCTATTGGGATGCACTATTAAACCTAAATTGGGGTTATCCGCTAAAAACTATGGTCGAGCAGTTTATGAATGTCTTCGCGGTGGATTATGCTCTTAACTCGACATCTTTTTCTCTGTTAACTCGAACCCGGTTTGTTGGGGTGTAATGGAATATGATGGAGCTCGAGTAGAAAGTATTGAGCTATTTA TCAAGGGA-----------------AAGGGGTCTAGGGTTAGTGTCAATCAAAAGAATAA

GTTGGAACAACTTCGTAAGTTATCTTTGACAGAAAAATAG-AAAGGATCAAAAAA

AAAATATAAAATTTTGAATCCCCCGGGACATTTTGATAAACCTTTGTT---AATTAA

TTTGCTTTATATATATCGTGCGGAAATCCCTCGTTCATATGATTAGATTCTTTGATAGAAATAA-TAACAAAAAGGTATGTTGCTGCCATTTTTGAAAGGATTAAAAATCA

ACGAAGTAATGTCTAAACCCAATGATTAAAAAAAAG--GATTT-AAAGGCTTCCGG

AACAAGGAAAGACTCTTTTTAATTGTCGCAACAATTGATTGGGATCAATTCAAAT

CGATGTTAAATGAGACAAAACAAAGGGTATTTTAGACTGCTCAATAAATAA-----------TAAATGC-----TAAAGGATTTTGCGGGGGGGGGGCTCCTTGAAACCGACCCAACT

TGAGTTATGAGTATACAAATGATTTTTT-----------TGAGGAAAGAA-----AAGAAAAG

GCTTAATTTTAATTCATTTGAGGATTGAGGATTTTATAGACTTTTTGATTGGTCAT

TCTAATTTATACATACATTTT--TTTTAATCATTTTT-CTCGAGCCGTACGAGGAGAA

AACAAAACTTTCAAATTCAGAGAAACCCTGGAATTAAAAAA--AGGGCAATCCTG

AGCCAACTCCCATTTTCTTTTTTTTTTTT--AAAGGAAAATAAAGGATTAAGAAAGC

AAGAAAA--AGGGGATAGGTGCAGAGACTCAAAGGGAGCTGTTCTAACAAATGGG

GTTGACTGTGTTATTATAAGTATAAGACTTCTTCCGTCTAAATTCCAAACCAA-----GAACAATAA-AAAGGGTGAAGAATATACGTACTGAAATTTTTAATGACAACCCGA

ATCTGTTCTGTATTTTTAATTT-TATTTTATTTTTTATATT-ATAGTAGAGATTTAGA

ATAGGGAATTAAAAATGCACGAATTGTTGTGAATCGATTCCAAGTTAAAAGCGGAATCCATATTTATT-CATCAAAACATTCACACTCACTCCATAATCTGATAGATCTT

GTGAAGAACTGATTAATCGGATGAGAATAAAGATAGAGTCCCGTTTTAGCATGTC

AA-TACTGATTAC

>Cen3 AAGTGTTGGATTTAAAGCTGGTGTTAAAGATTACAAATTGACTTATTATACTCCTGAGTATGAAACCCTAGATACTGATATCTTGGCAGCATTCCGAGTAACTCCACAACCCGGAGTTCCACCCGAAGAAGCAGGGGCCGCAGTAGCCGCCGAATCTTCTACGGGTACATGGACAACTGTATGGACCGACGGACTTACCAGTCTTGATCGTTACAAAGGACGATGCTACCACATCGAGCCTGTTGCTGGAGAGGAAAATCAATATATTTGTTATGTAGCTTACCCCTTAGACCTTTTTGAGGAAGGCTCTGTTACTAACATGTTTACTTCCATTGTGGGTAATGTATTTGGGTTCAAAGCCTTGCGTGCTCTACGTTTGGAGGATTTGCGAATCCCTGTTGCTTATGTAAAAACTTTCCTAGGCCCGCCTCACGGTATCCAAGTTGAAAGAGATAAATTGAACAAGTATGGCCGTCCCCTATTGGGATGCACTATTAAACCTAAATTGGGGTTATCCGCTAAAAACTATGGTCGAGCAGTTTATGAATGTCTTCGCGGTGGATTATGCTCTTAACTCGACATCTTTTTCTCTG-TTAACTCGAACCCGG

TTTGTTGGGGTGTAATGGAATATGATGGAGCTCGAGTAGAAAGTATTGAGCTATT

TATCAAGGGA-----------------AAGGGGTCTAGGGTTAGTGTCAATCAAAAGAATAAG

TTGGAACAACTTCGTAAGTTATCTTTGACAGAAAAATAG-AAAGGATCAAAAAAA

AAATATAAAATTTTGAATCCCCCGGGACATTTTGATAAACCTTTGTT---AATTAAT

TTGCTTTATATATATCGTGCGGAAATCCCTCGTTCATATGATTAGATTCTTTGATAGAAATAA-TAACAAAAAGGTATGTTGCTGCCATTTTTGAAAGGATTAAAAATCAA

CGAAGTAATGTCTAAACCCAATGATTAAAAAAAAG--GATTT-AAAGGCTTCCGGA

ACAAGGAAAGACTCTTTTTAATTGTCGCAACAATTGATTGGGATCAATTCAAATC

GATGTTAAATGAGACAAAACAAAGGGTATTTTAGACTGCTCAATAAATAA-----------TAAATGC-----TAAAGGATTTTGCGGGGGGGG--CTCCTTGAAACCGACCCAACTTG

AGTTATGAGTATACAAATGATTTTTT-----------TGAGGAAAGAA-----AAGAAAAGGC

TTAATTTTAATTCATTTGAGGATTGAGGATTTTATAGACTTTTTGATTGGTCATTCT

AATTTATACATACATTTT--TTTTAATCATTTTT-CTCGAGCCGTACGAGGAGAAAA

CAAAACTTTCAAATTCAGAGAAACCCTGGAATTAAAAAA--AGGGCAATCCTGAG

CCAACTCCCATTTTCTTTTTTTTTTTT--AAAGCAAAATAAAGGATTAAGAAAGCAA

GAAAA--AGGGGATAGGTGCAGAGACTCAAAGGGAGCTGTTCTAACAAATGGGGT

TGACTGTGTTATTATAAGTATAAGACTTCTTCCGTCTAAATTCCAAACCAA-----GA

ACAATAA-AAAGGGTGAAGAATATACGTACTGAAATTTTTAATGACAACCCGAAT

CTGTTCTGTATTTTTAATTT-TATTTTATTTTTTATATT-ATAGTAGAGATTTAGAAT

AGGGAATTAAAAATGCACGAATTGTTGTGAATCGATTCCAAGTTAAAAGCGGAA

TCCATATTTATT-CATCAAAACATTCACACTCACTCCATAATCTGATAGATCTTGTG

AAGAACTGATTAATCGGATGAGAATAAAGATAGAGTCCCGTTTTAGCATGTCAA-

TACTGATTAC

>Can3 AAGTGTTGGATTTAAAGCTGGTGTTAAAGATTACAAATTGACTTATTATACTCCTGAGTATGAAACCCTAGATACTGATATCTTGGCAGCATTCCGAGTAACTCCACAACCCGGAGTTCCACCCGAAGAAGCAGGGGCCGCAGTAGCCGCCGAATCTTCTACGGGTACATGGACAACTGTATGGACCGACGGACTTACCAGTCTTGATCGTTACAAAGGACGATGCTACCACATCGAGCCTGTTGCTGGAGAGGAAAATCAATATATTTGTTATGTAGCTTACCCCTTAGACCTTTTTGAGGAAGGCTCTGTTACTAACATGTTTACTTCCATTGTGGGTAATGTATTTGGGTTCAAAGCCTTGCGTGCTCTACGTTTGGAGGATTTGCGAATCCCTGTTGCTTATGTAAAAACTTTCCTAGGCCCGCCTCACGGTATCCAAGTTGAAAGAGATAAATTGAACAAGTATGGCCGTCCCCTATTGGGATGCACTATTAAACCTAAATTGGGGTTATCCGCTAAAAACTATGGTCGAGCAGTTTATGAATGTCTTCGCGGTGGATTATGCTCTTAACTCGACATCTTTTTCTCTG-TTAACTCGAACCCG

GTTTGTTGGGGTGTAATGGAATATGATGGAGCTCGAGTAGAAAGTATTGAGCTATTTATCAAGGGA-----------------AAGGGGTCTAGGGTTAGTGTCAATCAAAAGAATAA

GTTGGAACAACTTCGTAAGTTATCTTTGACAGAAAAATAG-AAAGGAT-AAAAAA

AAAATATAAAATTTTGAATCCCCCGGGACATTTTGATAAACCTTTGTT---AATTAA

TTTGCTTTATATATATCGTGCGGAAATCCCTCGTTCATATGATTAGATTCTTTGATAGAAATAA-TAACAAAAAGGTATGTTGCTGCCATTTTTGAAAGGATTAAAAATCA

ACGAAGTAATGTCTAAACCCAATGATTAAAAAAAAG--GATTT-AAAGGCTTCCGG

AACAAGGAAAGACTCTTTTTAATTGTCGCAACAATTGATTGGGATCAATTCAAAT

CGATGTTAAATGAGACAAAACAAAGGGTATTTTAGACTGCTCAATAAATAA-----------TAAATGC-----TAAAGGATTTTGCGGGGGGGG--CTTCTTGAAACCGACCCAACTT

GAGTTATGAGTATACAAATGATTTTTT-----------TGAGGAAAGAA-----AAGAAAAGG

CTTAATTTTAATTCATTTGAGGATTGAGGATTTTATAGACTTTTTGATTGGTCATTC

TAATTTATACATACATTTT--TTTTAATCTTTTTT-CTCGAGCCGTACGAGGAGAAAA

CAAAACTTTCAAATTCAGAGAAACCCTGGAATTAAAAAA--AGGGCAATCCTGAG

CCAACTCCCATTTTCTTTTTTTTTT--CAAAAGGAAAATAAAGGATTAAGAAAGCA

AGAAAA--AGGGGATAGGTGCAGAGACTCAAAGGGAGCTGTTCTAACAAATGGGG

TTGACTGTGTTATTATAAGTATAAGACTTCTTCCGTCTAAATTCCAAACCAA-----G

AACAATAA-AAAGGGTGAAGAATATACGTACTGAAATTTTTAATGACAACCCGAA

TCTGTTCTGTATTTTTAATTT-TATTTTATTTTTTATATT-ATAGTAGAGATTTAGAA

TAGGGAATTAAAAATGCACGAATTGTTGTGAATCGATTCCAAGTTAAAAGCGGA

ATCCATATTTATT-CATCAAAACATTCACACTCACTCCATAATCTGATAGATCTTGT

GAAGAACTGATTAATCGGATGAGAATAAAGATAGAGTCCCGTTTTAGCATGTCAA

-TACTGATTAC

>Bal2 AAGTGTTGGATTTAAAGCTGGTGTTAAAGATTACAAATTGACTTATTATACTCCTGAGTATGAAACCCTAGATACTGATATCTTGGCAGCATTCCGAGTAACTCCACAACCCGGAGTTCCACCCGAAGAAGCAGGGGCCGCAGTAGCCGCCGAATCTTCTACGGGTACATGGACAACTGTATGGACCGACGGACTTACCAGTCTTGATCGTTACAAAGGACGATGCTACCACATCGAGCCTGTTGCTGGAGAGGAAAATCAATATATTTGTTATGTAGCTTACCCCTTAGACCTTTTTGAGGAAGGCTCTGTTACTAACATGTTTACTTCCATTGTGGGTAATGTATTTGGGTTCAAAGCCTTGCGTGCTCTACGTTTGGAGGATTTGCGAATCCCTGTTGCTTATGTAAAAACTTTCCTAGGCCCGCCTCACGGTATCCAAGTTGAAAGAGATAAATTGAACAAGTATGGCCGTCCTCTATTGGGATGCACTATTAAACCTAAATTGGGGTTATCCGCTAAAAACTATGGTCGAGCAGTTTATGAATGTCTTCGCGGTGGATTATGCTCTTAACTCGACATCTTTTTATCTGTTTCACTCGAACCCGGTTTGTTGGGATGTAATGGAATATGATGGAGCTCGAGTAGAAAGTATTGAGTTATTTATCAAGGGA-----------------GAGGGGTCTAGGGTTAGTGTCAATCAAAAGAATCA

GTTGGAACAACTTCGTAAGTTATCTTTGACAGAAAAATAG-AAAGGATCAAAAAA

AAAATAAAAAATTTTGAATCCCCCGGGACATTTTGATAAACCTTTGTTGTTAATTA

ATTTGCTTTATATATATCGGGCGGAAATCCCTCGTTCATATGATTAGATTCTTTGATAGAAATAAATAACAAAAAGGTATGTTGCTGCTATTTTTGAAAGGATTAAAAATCAACGAAGTAATGTCTAAACCCAATGATTAAAAAAAAAAAGATTT-AAAGGCTTCC

GGAACAAGGAAAGACTCTTTTTAATTGTCGCAACAATTGATTGGGATCAATTCAA

ATCGATGTTAAATGAGACAAAACAAAGGGTATTTTAGACTGCTCAATAAATAA-----------TAAATGC-----TAAAGGATTTTGCGGGGGGGGGGCTCCTTGAAACCGACCCAA

CTTGAGTTATGAGTATACAAATGATTTTTT------------GAGGAAAGAA-----AAGAAA

AGGCTTAATTTTAATTCATTTGAGGATTGAGGATTTTATAGACTTTTTGATGGGTC

ATTCTAATTTATACATACATTTT---TTTAATCGTTTTTTCTCGAGCCGTACGAGGAG

AAAACAAAACTTTCAAATTCAGAGAAACCCTGGAATTAAAAAA-AGGGCAATCCT

GAGCCAACTCC-ATTTTCTTTTTTTTTTT-CAAAAGGAAAATAAAGGATTAAGAAA

GCAAGAAAA--AAGGGATAGGTGCAGAGACTCAAAGGGAGCTGTTCTAACAAATG

GGGTTGACTGTGTTATTATAAGTATAAGACTTCTTCCGTCGAAATTCCAAACCAA-----GAACAATAATAAAGGGTGAAGAATATACGTACTGAAATTTTTAATGACAACCC

GAATCTGTTCTGTATTTTTAATTT-TATTTTATTTTTTATATT-ATAGTAGAGATTTA

GAATAGGGAATTTAAAATGCACGAATTGTTGTGAATCGATTCTAAGTTAAAAGCG

GAATCCATATTTATT-CATCAAAACATTCACACTCACTCCATAATCTGATAGATCT

TGTGAAGAACTAATTAATCGGATGAGAATAAAGATAGAGTCCCGTTTTAGCATGT

CAA-TACTGATTAC

>Bal1 AAGTGTTGGATTTAAAGCTGGTGTTAAAGATTACAAATTGACTTATTATACTCCTGAGTATGAAACCCTAGATACTGATATCTTGGCAGCATTCCGAGTAACTCCACAACCCGGAGTTCCACCCGAAGAAGCAGGGGCCGCAGTAGCCGCCGAATCTTCTACGGGTACATGGACAACTGTATGGACCGACGGACTTACCAGTCTTGATCGTTACAAAGGACGATGCTACCACATCGAGCCTGTTGCTGGAGAGGAAAATCAATATATTTGTTATGTAGCTTACCCCTTAGACCTTTTTGAGGAAGGCTCTGTTACTAACATGTTTACTTCCATTGTGGGTAATGTATTTGGGTTCAAAGCCTTGCGTGCTCTACGTTTGGAGGATTTGCGAATCCCTGTTGCTTATGTAAAAACTTTCCTAGGCCCGCCTCACGGTATCCAAGTTGAAAGAGATAAATTGAACAAGTATGGCCGTCCTCTATTGGGATGCACTATTAAACCTAAATTGGGGTTATCCGCTAAAAACTATGGTCGAGCAGTTTATGAATGTCTTCGCGGTGGATTATGCTCTTAACTCGACATCTTTTTATCTGTTTCACTCGAACCCGGTTTGTTGGGATGTAATGGAATATGATGGAGCTCGAGTAGAAAGTATTGAGTTATTTATCAAGGGA-----------------GAGGGGTCTAGGGTTAGTGTCAATCAAAAGAATCA

GTTGGAACAACTTCGTAAGTTATCTTTGACAGAAAAATAG-AAAGGAT-AAAAAA

AAAATATAAAATTTTGAATCCCCCGGGACATTTTGATAAACCTTTGTTGTTAATTA

ATTTGCTTTATATATATCGGGCGGAAATCCCTCGTTCATATGATTAGATTCTTTGATAGAAATAAATAACAAAAAGGTATGTTGCTGCTATTTTTGAAAGGATTAAAAATCAACGAAGTAATGTCTAAACCCAATGATTAAAAAAAAAAAGATTT-AAAGGCTTCC

GGAACAAGGAAAGACTCTTTTTAATTGTCGCAACAATTGATTGGGATCAATTCAA

ATCGATGTTAAATGAGACAAAACAAAGGGTATTTTAGACTGCTCAATAAATAAA-------TAATAAATGC-----TAAAGGATTTTGCGGGGGGGGGGCTCCTTGAAACCGACCC

AACTTGAGTTATGAGTATACAAATGATTTTTT------------GAGGAAAGAA-----AAGA

AAAGGCTTAATTTTAATTCATTTGAGGATTGAGGATTTTATAGACTTTTTGATTGG

TCATTCTAAGTTATACATACATTTT---TTTAATCGTTTTTTCTCGAGCCGTACGAGG

AGAAAACAAAACTTTCAAATTCAGAGAAACCCTGGAATTAAAAAA--AGGGCAAT

CCTGAGCCAACTCC-ATTTTCTTTTTTTTTTT-CAAAAGGAAAATAAAGGATTAAGA

AAGCAAGAAAA--AAGGGATAGGTGCAGAGACTCAAAGGGAGCTGTTCTAACAAA

TGGGGTTGACTGTGTTATTATAAGTATAAGACTTCTTCCGTCGAAATTCCAAACCAA-----GAACAATAATAAAGGGTGAAGAATATACGTACTGAAATTTTTAATGACAAC

CCGAATCTGTTCTGTATTTTTAATTT-TATTTTATTTTTTATATT-ATAGTAGAGATT

TAGAATAGGGAATTTAAAATGCACGAATTGTTGTGAATCGATTCTAAGTTAAAAG

CGGAATCCATATTTATT-CATCAAAACATTCACACTCACTCCATAATCTGATAGAT

CTTGTGAAGAACTGATTAATCGGATGAGAATAAAGATAGAGTCCCGTTTTAGCAT

GTCAA-TACTGATTAC

>Ibe2 AAGTGTTGGATTTAAAGCTGGTGTTAAAGATTACAAATTGACTTATTATACTCCTGAGTATGAAACCCTAGATACTGATATCTTGGCAGCATTCCGAGTAACTCCACAACCCGGAGTTCCACCCGAAGAAGCAGGGGCCGCAGTAGCCGCCGAATCTTCTACGGGTACATGGACAACTGTATGGACCGACGGACTTACCAGTCTTGATCGTTACAAAGGACGATGCTACCACATCGAGCCTGTTGCTGGAGAGGAAAATCAATATATTTGTTATGTAGCTTACCCCTTAGACCTTTTTGAGGAAGGCTCTGTTACTAACATGTTTACTTCCATTGTGGGTAATGTATTTGGGTTCAAAGCCTTGCGTGCTCTACGTTTGGAGGATTTGCGAATCCCTGTTGCTTATGTAAAAACTTTCCTAGGCCCGCCTCACGGTATCCAAGTTGAAAGAGATAAATTGAACAAGTATGGCCGTCCCCTATTGGGATGCACTATTAAACCTAAATTGGGGTTATCCGCTAAAAACTATGGTCGAGCAGTTTATGAATGTCTTCGCGGTGGATTATGCTCTTAACTCGACATCTTTTTCTCTG-TTAACTCGAACCTG

GTTTGTTGGGGTGTAATGGAATATGATGGAGCTCGAGTAGAAAGTATTGAGCTATTTATCAAGGGA-----------------AAGGGGTCTAGGGTTAGTGTCAATCAAAAGAATAA

GTTGGAACAACTTCGTAAGTTATCTTTGACAGAAAAATAG-AAAGGATC-AAAAA

AAAATATAAAATTTTGAATCCCCCGGGACATTTTGATAAACCTTTGTT---AATTAA

TTTGCTTTATATATATCGTGCGGAAATCCCTCGTTCATATGATTAGATTCTTTGATAGAAATAA-TAACAAAAAGGTATGTTGCTGCCATTTTTGAAAGGATTAAAAATCA

ACGAAGTAATGTCTAAACCCAATGATTAAAAAAAAAG-GATTTTAAAGGTTTCCG

GAACAAGGAAAGACTCTTTTTAATTGTCGCAACAATTGATTGGGATCAATTCAAA

TCGATGTTAAATGAGACAAAACAAAGGGTATTTTAGACTGCTCAATAAATAA-----------TAAATGC-----TAAAGGATTTTGCGGGGGGGGG-CTCCTTGAAACCGACCCAAC

TTGAGTTATGAGTATACAAATGATTTTTT-----------TGAGGAAAGAA-----AAGAAAA

GGCTTAATTTTAATTCATTTGAGGATTGAGGATTTTATAGACTTTTTGATTGGTCATTCTAATTTATACATACATTTT--TTTT-ATCATTTTT-CTCGAGCCGTACGAGGAGA

AAACAAAACTTTCAAATTCAGAGAAACCCTGGAATTAAAAAA--AGGGCAATCCT

GAGCCAACTCC-ATTTTCTTTTTTTTTTT-CAAAAGGAAAATAAAGGATTAAGAAA

GCAAGAAAA--AAGGGATAGGTGCAGAGACTCAAAGGGAGCTGTTCTAACAAATG

GGGTTGACTGTGTTATTATAAGTATAAGACTTCTTCCGTCGAAATTCCAAACCAA-----GAACAATAATAAAGGGTGAAGAATATACGTACTGAAATTTTTAATGACAACCC

GAATCTGTTCTGTATTTTTAATTT-TATTTTATTTTTTATATT-ATAGTAGAGATTTA

GAATAGGGAATTTAAAATGCACGAATTGTTGTGAATCGATTCTAAGTTAAAAGCG

GAATCCATATTTATT-CATCAAAACATTCACACTCACTCCATAATCTGATAGATCT

TGTGAAGAACTAATTAATCGGATGAGAATAAAGATAGAGTCCCGTTTTAGCATGTCAA-TACTGATTAC

>Can1 AAGTGTTGGATTTAAAGCTGGTGTTAAAGATTACAAATTGACTTATTATACTCCTGAGTATGAAACCCTAGATACTGATATCTTGGCAGCATTCCGAGTAACTCCACAACCCGGAGTTCCACCCGAAGAAGCAGGGGCCGCAGTAGCCGCCGAATCTTCTACGGGTACATGGACAACTGTATGGACCGACGGACTTACCAGTCTTGATCGTTACAAAGGACGATGCTACCACATCGAGCCTGTTGCTGGAGAGGAAAATCAATATATTTGTTATGTAGCTTACCCCTTAGACCTTTTTGAGGAAGGCTCTGTTACTAACATGTTTACTTCCATTGTGGGTAATGTATTTGGGTTCAAAGCCTTGCGTGCTCTACGTTTGGAGGATTTGCGAATCCCTGTTGCTTATGTAAAAACTTTCCTAGGCCCGCCTCACGGTATCCAAGTTGAAAGAGATAAATTGAACAAGTATGGCCGTCCCCTATTGGGATGCACTATTAAACCTAAATTGGGGTTATCCGCTAAAAACTATGGTCGAGCAGTTTATGAATGTCTTCGCGGTGGATTATGCTCTTAACTCGACATCTTTTTCTCTG-TTAACTCGAACCCGG

TTTTTTGGGGTGTAATGGAATATGATGGAGCTCGAGTAGAAAGTATTGAGCTATTTATCAAGGGA-----------------AAGGGGTCTAGGGTTAGTGTCAATCAAAAGAATAAG

TTGGAACAACTTCGTAAGTTATCTTTGACAGAAAAATAG-AAAGGATCAAAAAAA

AAATATAAAATTTTGAATCCCCCGGGACATTTTGATAAACCTTTGTT---AATTAAT

TTGCTTTATATATATCGTGCGGAAATCCCTCGTTCATATGATTAGATTCTTTGATAGAAATAA-TAACAAAAAGGTATGTTGCTGCCATTTTTGAAAGGATTAAAAATCAA

CGAAGTAATGTCTAAACCCAATGATTAAAAAAAAA--GATTT-AAAGGCTTCCGGA

ACAAGGAAAGACTCTTTTTAATTGTCGCAACAATTGATTGGGATCAATTCAAATCGATGTTAAATGAGACAAAACAAAGGGTATTTTAGACTGCTCAATAAATAA----------

-TAAATGC-----TAAAGGATTTTGCGGGGGGGG--CTCCTTGAAACCGACCCAACTTG

AGTTATGAGTATACAAATGATTTTTT-----------TGAGGAAAGAA-----AAGAAAAGGC

TTAATTTTAATTCATTTGAGGATTGAGGATTTTATAGACTTTTTGATTGGTCATTCTAATTTATACATACATTTT--TTTTAATCATTTTT-CTCGAGCCGTACGAGGAGAAAA

CAAAACTTTCAAATTCAGAGAAACCCTGGAATTAAAAAA--AGGGCAATCCTGAG

CCAACTCC-ATTTTCTTTTTTTTTTT-CAAAAGGAAAATAAAGGATTAAGAAAGCA

AGAAAA--AAGGGATAGGTGCAGAGACTCAAAGGGAGCTGTTCTAACAAATGGGG

TTGACTGTGTTATTATAAGTATAAGACTTCTTCCGTCGAAATTCCAAACCAA-----G

AACAATAATAAAGGGTGAAGAATATACGTACTGAAATTTTTAATGACAACCCGA

ATCTGTTCTGTATTTTTAATTT-TATTTTATTTTTTATATT-ATAGTAGAGATTTAGA

ATAGGGAATTTAAAATGCACGAATTGTTGTGAATCGATTCTAAGTTAAAAGCGGA

ATCCATATTTATT-CATCAAAACATTCACACTCACTCCATAATCTGATAAATCTTGT

GAAGAACTGATTAATCGGATGAGAATAAAGATAGAGTCCCGTTTTAGCATGTCAA

-TACTGATTAC

>Can2 AAGTGTTGGATTTAAAGCTGGTGTTAAAGATTACAAATTGACTTATTATACTCCTGAGTATGAAACCCTAGATACTGATATCTTGGCAGCATTCCGAGTAACTCCACAACCCGGAGTTCCACCCGAAGAAGCAGGGGCCGCAGTAGCCGCCGAATCTTCTACGGGTACATGGACAACTGTATGGACCGACGGACTTACCAGTCTTGATCGTTACAAAGGACGATGCTACCACATCGAGCCTGTTGCTGGAGAGGAAAATCAATATATTTGTTATGTAGCTTACCCCTTAGACCTTTTTGAGGAAGGCTCTGTTACTAACATGTTTACTTCCATTGTGGGTAATGTATTTGGGTTCAAAGCCTTGCGTGCTCTACGTTTGGAGGATTTGCGAATCCCTGTTGCTTATGTAAAAACTTTCCTAGGCCCGCCTCACGGTATCCAAGTTGAAAGAGATAAATTGAACAAGTATGGCCGTCCCCTATTGGGATGCACTATTAAACCTAAATTGGGGTTATCCGCTAAAAACTATGGTCGAGCAGTTTATGAATGTCTTCGCGGTGGATTATGCTCTTAACTCGACATCTTTTTCTCTG-TTAACTCGAACCCG

GTTTGTTGGGGTGTAATGGAATATGATGGAGCTCGAGTAGAAAGTATTGAGCTATTTATCAAGGGA-----------------AAGGGGTCTAGGGTTAGTGTCAATCAAAAGAATAA

GTTGGAACAACTTCGTAAGTTATCTTTGACAGAAAAATAG-AAAGGAT--AAAAAA

AAATATAAAATTTTGAATCCCCCGGGACATTTTGATAAACCTTTGTT---AATTAAT

TTGCTTTATATATATCGTGCGGAAATCCCTCGTTCATATGATTAGATTCTTTGATAGAAATAA-TAACAAAAAGGTATGTTGCTGCCATTTTTGAAAGGATTAAAAATCAA

CGAAGTAATGTCTAAACCCAATGATTAAAAAAAAG--GATTT-AAAGGCTTCCGGA

ACAAGGAAAGACTCTTTTTAATTGTCGCAACAATTGATTGGGATCAATTCAAATCGATGTTAAATGAGACAAAACAAAGGGTATTTTAGACTGCTCAATAAATAA-----------TAAATGC-----TAAAGGATTTTGCGGGGGGGG--CTCCTTGAAACCGACCCAACTTG

AGTTATGAGTATACAAATGATTTTTT-----------TGAGGAAAGAA-----AAGAAAAGGC

TTAATTTTAATTCATTTGAGGATTGAGGATTTTATAGACTTTTTGATTGGTCATTCT

AATTTATACATACATTTT--TTTTAATCTTTTTT-CTCGAGCCGTACGAGGAGAAAAC

AAAACTTTCAAATTCAGAGAAACCCTGGAATTAAAAAA--AGGGCAATCCTGAGC

CAACTCC-ATTTTCTTTTTTTTTT--CAAAAGGAAAATAAAGGATTAAGAAAGCAAG

AAAA--AAGGGATAGGTGCAGAGACTCAAAGGGAGCTGTTCTAACAAATGGGGTT

GACTGTGTTATTATAAGTATAAGACTTCTTCCGTCGAAATTCCAAACCAA-----GAA

CAATAATAAAGGGTGAAGAATATACGTACTGAAATTTTTAATGACAACCCGAATCTGTTCTGTATTTTTAATTT-TATTTTAATATTTATATT-ATAGTAGAGATTTAGAATA

GGGAATTTAAAATGCACGAATTGTTGTGAATCGATTCTAAGTTAAAAGCGGAATC

CATATTTATT-CATCAAAACATTCACACTCACTCCATAATCTGATAGATCTTGTGA

AGAACTAATTAATCGGATGAGAATAAAGATAGAGTCCCGTTTTAGCATGTCAA-T

ACTGATTAC

>Mas AAGTGTTGGATTTAAAGCTGGTGTTAAAGATTACAAATTGACTTATTATACCCCTGAGTATGAAACCCTAGATACTGATATCTTGGCAGCATTCCGAGTAACTCCACAACCCGGAGTTCCACCCGAAGAAGCAGGGGCCGCAGTAGCCGCCGAATCTTCTACGGGTACATGGACAACTGTATGGACCGACGGACTTACCAGTCTTGATCGTTACAAAGGACGATGCTACCACATCGAGCCTGTTGCTGGAGAGGAAAATCAATATATTTGTTATGTAGCTTACCCCTTAGACCTTTTTGAGGAAGGCTCTGTTACTAACATGTTTACTTCCATTGTGGGTAATGTATTTGGGTTCAAAGCCTTGCGTGCTCTACGTTTGGAGGATTTGCGAATCCCTGTTGCTTATGTAAAAACTTTCCTAGGCCCGCCTCACGGTATCCAAGTTGAAAGAGATAAATTGAACAAGTATGGCCGTCCCCTATTGGGATGCACTATTAAACCTAAATTGGGGTTATCCGCTAAAAACTATGGTCGAGCAGTTTATGAATGTCTTCGCGGTGGATTGTGCTCTTAACTCGACATCTTTTTCTCTG-TTAACTCGAACCCG

GTTTGTTGGGGTGTAATGGAATATGATGGAGCTCGAGTAGAAAGTATTGAGCTATTTATCAAGGGA-----------------AAGGGGTCTAGGGTTAGTGTCAATCAAAAGAATAA

GTTGGAACAACTTCGTAAGTTATCTTTGACAGAAAAATAG-AAAGGATC-AAAAA

AAAATATAAAATTTTGAATCCCCCGGGACATTTTGATAAACCTTTGTT---AATTA

ATTTGCTTTATATATATCGTGCGGAAATCCCTTGTTCATATGATTAGATTCTTTGATAGAAATAAATAACAAAAAGGTATGTTGCTGCCATTTTTGAAAGGATTAAAAATC

AACGAAGTAATGTCTAAACCCAATGATTAAAAAAAAAAGGATTTAAAGGCTTCCGGAACAAGGAAAGACTCTTTTTAATTGTCGCAACAATTGATTGGGATCAATTCAA

ATCGATGTTAAATGAGACAAAACAAAGGGTATTTTAGACTGCTCAATAAATAA-----------TAAATGC-----TAAAGGATTTTGCGGGGGGGGG-CTCCTTGAAACCGACCCAA

CTTGAGTTATGAGTATACAAATGATTTTTT-----------TGAGGAAAGAA-----AAGAAA

AGGCTTAATTTTAATTCATTTGAGGATTGAGGATTTTATAGACTTTTTGATTGGTCATTCTAATTTATACATACATTTT--TTTTAATCATTTTT-CTCGAGCCGTACGAGGAG

AAAACAAAACTTTCAAATTCAGAGAAACCCTGGAATTAAAAAA--AGGGCAATCC

TGAGCCAACTCC-ATTTTCTTTTTTTTTTT-CAAAAGGAAAATAAAGGATTAAGAAA

GCAAGAAAA--AAGGGATAGGTGCAGAGACTCAAAGGGAGCTGTTCTAACAAATG

GGGTTGACTGTGTTATTATAAGTATAAGACTTCTTCCGTCGAAATTCCAAACCAA-----GAACAATAATAAAGGGTGAAGAATATACGTACTGAAATTTTTAATGACAACCC

GAATCTGTTCTGTATTTTTAATTT-TATTTTATTTTTTATATT-ATAGTAGAGATTTA

GAATAGGGAATTTAAAATGCACGAATTGTTGTGAATCGATTCTAAGTTAAAAGCGGAATCCATATTTATT-CATCAAAACATTCACACTCACTCCATAATCTGATAGATCT

TGTGAAGAACTAATTAATCGGATGAGAATAAAGATAGAGTCCCGTTTTAGCATGT

CAA-TACTGATTAC

>Pyr1 AAGTGTTGGATTTAAAGCTGGTGTTAAAGATTACAAATTGACTTATTATACTCCTGAGTATGAAACCCTAGATACTGATATCTTGGCAGCATTCCGAGTAACTCCACAACCCGGAGTTCCACCCGAAGAAGCAGGGGCCGCAGTAGCCGCCGAATCTTCTACGGGTACATGGACAACTGTATGGACCGACGGACTTACCAGTCTTGATCGTTACAAAGGACGATGCTACCACATCGAGCCTGTTGCTGGAGAGGAAAATCAATATATTTGTTATGTAGCTTACCCCTTAGACCTTTTTGAGGAAGGCTCTGTTACTAACATGTTTACTTCCATTGTGGGTAATGTATTTGGGTTCAAAGCCTTGCGTGCTCTACGTTTGGAGGATTTGCGAATCCCTGTTGCTTATGTAAAAACTTTCCTAGGCCCGCCTCACGGTATCCAAGTTGAAAGAGATAAATTGAACAAGTATGGCCGTCCCCTATTGGGATGCACTATTAAACCTAAATTGGGGTTATCCGCTAAAAACTATGGTCGAGCAGTTTATGAATGTCTTCGCGGTGGATTATGCTCTTAACTCGACATCTTTTTCTCTG-TTAACTCGAACCCGG

TTTGTTGGGGTGTAATGGAATATGATGGAGCTCGAGTAGAAAGTATTGAGCTATTTATCAAGGGA-----------------AAGGGGTCTAGGGTTAGTGTCAATCAAAAGAATAAG

TTGGAACAACTTCGTAAGTTATCTTTGACAGAAAAATAG-AAAGGAT--AAAAAAA

AATATAAAATTTTGAATCCCCCGGGACATTTTGATAAACCTTTGTT---AATTAATT

TGCTTTATATATATCGTGCGGAAATCCCTCGTTCATATGATTAGATTCTTTGATAGAAATAA-TAACAAAAAGGTATGTTGCTGCCATTTTTGAAAGGATTAAAAATCAAC

GAAGTAATGTCTAAACCCAATGATTAAAAAAAAG--GATTT-AAAGGCTTCCGGAA

CAAGGAAAGACTCTTTTTAATTGTCGCAACAATTGATTGGGATCAATTCAAATCG

ATGTTAAATGAGACAAAACAAAGGGTATTTTAGACTGCTCAATAAATAA-----------TAAATGC-----TAAAGGATTTTGCGGGGGGGG--CTCCTTGAAACCGACCCAACTTG

AGTTATGAGTATACAAATGATTTTTT-----------TGAGGAAAGAA-----AAGAAAAGGC

TTAATTTTAATTCATTTGAGGATTGAGGATTTTATAGACTTTTTGATTGGTCATTCTAATTTATACATACATTTT--TTTTAATCTTTTTT-CTCGAGCCGTACGAGGAGAAAAC

AAAACTTTCAAATTCAGAGAAACCCTGGAATTAAAAAAAAAGGGCAATCCTGAGCCAACTCC-ATTTTCTTTTTTTTTT--CAAAAGCAAAAAAAAAGATTAAGAAAGCAA

GAATAAAAAAGGATAGGTGCAGAGACTCAAAGGGAGCTATTCTAACAAATGGGGTTGACTGTGTTGTTATAAGTATAAGGCTTCTTCCGTCTAAATTCCAAATCCAATACAAACCAAGAATAAAGGATGAAGAATATACGTACTGAAATGATTAATGACAACCCGAATCTGTTCTGTATTTTTTTTTT-TTTTTT----------TT-ATAGTAGAGATTTAGAATA

GGAAATGAAAAATGCAATAATTGTTGTGAATCGATTCGAAGTTAAAAGCGGAAT

CCATATTTATT-CATCAAAACATTCACACTCACTCCATAGTCTGATAGATCTTGTG

AAGAACTGATTAATCGGATGAGAATAAAGATAGAGTCCCGTTCTA-CATGTCAA-T

ACTGACAAC

>Pyr2 AAGTGTTGGATTTAAAGCTGGTGTTAAAGATTACAAATTGACTTATTATACTCCTGAGTATGAAACCCTAGATACTGATATCTTGGCAGCATTCCGAGTAACTCCACAACCCGGAGTTCCACCCGAAGAAGCAGGGGCCGCAGTAGCCGCCGAATCTTCTACGGGTACATGGACAACTGTATGGACCGACGGACTTACCAGTCTTGATCGTTACAAAGGACGATGCTACCACATCGAGCCTGTTGCTGGAGAGGAAAATCAATATATTTGTTATGTAGCTTACCCCTTAGACCTTTTTGAGGAAGGCTCTGTTACTAACATGTTTACTTCCATTGTGGGTAATGTATTTGGGTTCAAAGCCTTGCGTGCTCTACGTTTGGAGGATTTGCGAATCCCTGTTGCTTATGTAAAAACTTTCCTAGGCCCGCCTCACGGTATCCAAGTTGAAAGAGATAAATTGAACAAGTATGGCCGTCCCCTATTGGGATGCACTATTAAACCTAAATTGGGGTTATCCGCTAAAAACTATGGTCGAGCAGTTTATGAATGTCTTCGCGGTGGATTATGCTCTTAACTCGACATCTTTTTCTCTG-TTAACTCGAACCCGG

TTTGTTGGGGTGTAATGGAATATGATGGAGCTCGAGTAGAAAGTATTGAGCTATTTATCAAGGGA-----------------AAGGGGTCTAGGGTTAGTGTCAATCAAAAGAATAAG

TTGGAACAACTTCGTAAGTTATCTTTGACAGAAAAATAG-AAAGGATC-AAAAAA

AAATATAAAATTTTGAATCCCCCGGGACATTTTGATAAACCTTTGTT---AATTAAT

TTGCTTTATATATATCGTGCGGAAATCCCTCGTTCATATGATTAGATTCTTTGATAGAAATAA-TAACAAAAAGGTATGTTGCTGCCATTTTTGAAAGGATTAAAAATCAA

CGAAGTAATGTCTAAACCCAATGATTAAAAAAAAG--GATTT-AAAGGCTTCCGGA

ACAAGGAAAGACTCTTTTTAATTGTCGCAACAATTGATTGGGATCAATTCAAATC

GATGTTAAATGAGACAAAACAAAGGGTATTTTAGACTGCTCAATAAATAA-----------TAAATGC-----TAAAGGATTTTGCGGGGGGGG--CTCCTTGAAACCGACCCAACTTG

AGTTATGAGTATACAAATGATTTTTT-----------TGAGGAAAGAA-----AAGAAAAGGC

TTAATTTTAATTCATTTGAGGATTGAGGATTTTATAGACTTTTTGATTGGTCATTCT

AATTTATACATACATTTT--TTTTAATCATTTTT-CTCGAGCCGTACGAGGAGAAAA

CAAAACTTTCAAATTCAGAGAAACCCTGGAATTAAAAAA--AGGGCAATCCTGAG

CCAACTCCCATTTTCTTTTTTTTTTT-CAAAAGGAAAATAAAGGATTAAGAAAGCA

AGAAAA--AGGGGATAGGTGCAGAGACTCAAAGGGAGCTGTTCTAACAAATGGGG

TTGACTGTGTTATTATAAGTATAAGACTTCTTCCGTCTAAATTCCAAACCAA-----G

AACAATAA-AAAGGGTGAAGAATATACGTACTGAAATTTTTAATGACAACCCGAA

TCTGTTCTGTATTTTTAATTT-TATTTTATTTTTTATATT-ATAGTAGAGATTTAGAA

TAGGGAATTAAAAATGCACGAATTGCTGTGAATCGATTCCAAGTTAAAAGCGGA

ATCCATATTTATT-CATCAAAACATTCACACTCACTCCATAATCTGATAGATCTTGT

GAAGAACTGATTAATCGGATGAGAATAAAGATAGAGTCCCGTTTTAGCATGTCAA

-TACTGATTAC

>Pyr3 AAGTGTTGGATTTAAAGCTGGTGTTAAAGATTACAAATTGACTTATTATACCCCTGAGTATGAAACCCTAGATACTGATATCTTGGCAGCATTCCGAGTAACTCCACAACCCGGAGTTCCACCCGAAGAAGCAGGGGCCGCAGTAGCCGCCGAATCTTCTACGGGTACATGGACAACTGTATGGACCGACGGACTTACCAGTCTTGATCGTTACAAAGGACGATGCTACCACATCGAGCCTGTTGCTGGAGAGGAAAATCAATATATTTGTTATGTAGCTTACCCCTTAGACCTTTTTGAGGAAGGCTCTGTTACTAACATGTTTACTTCCATTGTGGGTAATGTATTTGGGTTCAAAGCCTTGCGTGCTCTACGTTTGGAGGATTTGCGAATCCCTGTTGCTTATGTAAAAACTTTCCTAGGCCCGCCTCACGGTATCCAAGTTGAAAGAGATAAATTGAACAAGTATGGCCGTCCCCTATTGGGATGCACTATTAAACCTAAATTGGGGTTATCCGCTAAAAACTATGGTCGAGCAGTTTATGAATGTCTTCGCGGTGGATTGTGCTCTTAACTCGACATCTTTTTCTCTG-TTAACTCGAACCCGG

TTTGTTGGGGTGTAATGGAATATGATGGAGCTCGAGTAGAAAGTATTGAGCTATT

TATCAAGGGA-----------------AAGGGGTCTAGGGTTAGTGTCAATCAAAAGAATAAG

TTGGAACAACTTCGTAAGTTATCTTTGACAGAAAAATAG-AAAGGATC-AAAAAA

AAATATAAAATTTTGAATCCCCCGGGACATTTTGATAAACCTTTGTT---AATTAAT

TTGCTTTATATATATCGTGCGGAAATCCCTTGTTCATATGATTAGATTCTTTGATAGAAATAAATAACAAAAAGGTATGTTGCTGCCATTTTTGAAAGGATTAAAAATCAA

CGAAGTAATGTCTAAACCCAATGATTAAAAAAAAAAGGATTT-AAAGGCTTCCGG

AACAAGGAAAGACTCTTTTTAATTGTCGCAACAATTGATTGGGATCAATTCAAAT

CGATGTTAAATGAGACAAAACAAAGGGTATTTTAGACTGCTCAATAAATAA-----------TAAATGC-----TAAAGGATTTTGCGGGGGGGG--CTCCTTGAAACCGACCCAACTT

GAGTTATGAGTATACAAATGATTTTTT-----------TGAGGAAAGAA-----AAGAAAAGG

CTTAATTTTAATTCATTTGAGGATTGAGGATTTTATAGACTTTTTGATTGGTCATTC

TAATTTATACATACATTTT--TTTTAATCATTTTT-CTCGAGCCGTACGAGGAGAAA

ACAAAACTTTCAAATTCAGAGAAACCCTGGAATTAAAAAA--AGGGCAATCCTGA

GCCAACTCC-ATTTTCTTTTTTTTTTT-CAAAAGGAAAATAAAGGATTAAGAAAGC

AAGAAAA--AGGGGATAGGTGCAGAGACTCAAAGGGAGCTGTTCTAAAAAATGGG

GTTGACTGTGTTATTATAAGTATAAGACTTCTTCCGTCTAAATTCCAAACCAA-----

GAACAATAA-AAAGGGTGAAGAATATACGTACTGAAATTTTTAATGACAACCCGA

ATCTGTTCTGTATTTTTAATTT-TATTTTATTTTTTATATT-ATAGTAGAGATTTAGA

ATAGGGAATTTAAAATGCACGAATTGTTGTGAATCGATTCCAAGTTAAAAGCGGA

ATCCATATTTATT-CATCAAAACATTCACACTCACTCCATAATCTGATAGATCTTGT

GAAGAACTGATTAATCGGATGAGAATAAAGATAGAGTCCCGTTTTA-CATGTCAA-TACTGACAAC

>Pyr5 AAGTGTTGGATTTAAAGCTGGTGTTAAAGATTACAAATTGACTTATTATACCCCTGAGTATGAAACCCTAGATACTGATATCTTGGCAGCATTCCGAGTAACTCCACAACCCGGAGTTCCACCCGAAGAAGCAGGGGCCGCAGTAGCCGCCGAATCTTCTACGGGTACATGGACAACTGTATGGACCGACGGACTTACCAGTCTTGATCGTTACAAAGGACGATGCTACCACATCGAGCCTGTTGCTGGAGAGGAAAATCAATATATTTGTTATGTAGCTTACCCCTTAGACCTTTTTGAGGAAGGCTCTGTTACTAACATGTTTACTTCCATTGTGGGTAATGTATTTGGGTTCAAAGCCTTGCGTGCTCTACGTTTGGAGGATTTGCGAATCCCTGTTGCTTATGTAAAAACTTTCCTAGGCCCGCCTCACGGTATCCAAGTTGAAAGAGATAAATTGAACAAGTATGGCCGTCCCCTATTGGGATGCACTATTAAACCTAAATTGGGGTTATCCGCTAAAAACTATGGTCGAGCAGTTTATGAATGTCTTCGCGGTGGATTGTGCTCTTAACTCGACATCTTTTTCTCTG-TTAACTCGAACCCGG

TTTGTTGGGGTGTAATGGAATATGATGGAGCTCGAGTAGAAAGTATTGAGCTATTTATCAAGGGA-----------------AAGGGGTCTAGGGTTAGTGTCAATCAAAAGAATAAG

TTGGAACAACTTCGTAAGTTATCTTTGACAGAAAAATAG-AAAGGATC-AAAAAA

AAATATAAAATTTTGAATCCCCCGGGACATTTTGATAAACCTTTGTT---AATTAA

TTTGCTTTATATATATCGTGCGGAAATCCCTTGTTCATATGATTAGATTCTTTGATAGAAATAAATAACAAAAAGGTATGTTGCTGCCATTTTTGAAAGGATTAAAAATCA

ACGAAGTAATGTCTAAACCCAATGATTAAAAAAAAAAAGATTT-AAAGGCTTCCG

GAACAAGGAAAGACTCTTTTTAATTGTCGCAACAATTGATTGGGATCAATTCAAA

TCGATGTTAAATGAGACAAAACAAAGGGTATTTTAGACTGCTCAATAAATAA-----------TAAATGC-----TAAAGGATTTTGCGGGGGGGGG-CTCCTTGAAACCGACCCAAC

TTGAGTTATGAGTATACAAATGATTTTTT-----------TGAGGAAAGAA-----AAGAAAA

GGCTTAATTTTAATTCATTTGAGGATTGAGGATTTTATAGACTTTTTGATTGGTCATTCTAATTTATACATACATTTT--TTTTAATCATTTTT-CTCGAGCCGTACGAGGAGA

AAACAAAACTTTCAAATTCAGAGAAACCCTGGAATTAAAAAA--AGGGCAATCCT

GAGCCAACTCC-ATTTTCTTTTTTTTTTT-CAAAAGGAAAATAAAGGATTAAGAAA

GCAAGAAAA--AGGGGATAGGTGCAGAGACTCAAAGGGAGCTGTTCTAAAAAATG

GGGTTGACTGTGTTATTATAAGTATAAGACTTCTTCCGTCTAAATTCCAAACCAA-----GAACAATAA-AAAGGGTGAAGAATATACGTACTGAAATTTTTAATGACAACCC

GAATCTGTTCTGTATTTTTAATTT-TATTTTATTTTTTATATT-ATAGTAGAGATTTA

GAATAGGGAATTTAAAATGCACGAATTGTTGTGAATCGATTCCAAGTTAAAAGCG

GAATCCATATTTATT-CATCAAAACATTCACACTCACTCCATAATCTGATAGATCT

TGTGAAGAACTGATTAATCGGATGAGAATAAAGATAGAGTCCCGTTTTA-CATGT

CAA-TACTGACAAC

>Ibe1 AAGTGTTGGATTTAAAGCTGGTGTTAAAGATTACAAATTGACTTATTATACTCCTGAGTATGAAACCCTAGATACTGATATCTTGGCAGCATTCCGAGTAACTCCACAACCCGGAGTTCCACCCGAAGAAGCAGGGGCCGCAGTAGCCGCCGAATCTTCTACGGGTACATGGACAACTGTATGGACCGACGGACTTACCAGTCTTGATCGTTACAAAGGACGATGCTACCACATCGAGCCTGTTGCTGGAGAGGAAAATCAATATATTTGTTATGTAGCTTACCCCTTAGACCTTTTTGAGGAAGGCTCTGTTACTAACATGTTTACTTCCATTGTGGGTAATGTATTTGGGTTCAAAGCCTTGCGTGCTCTACGTTTGGAGGATTTGCGAATCCCTGTTGCTTATGTAAAAACTTTCCTAGGCCCGCCTCACGGTATCCAAGTTGAAAGAGATAAATTGAACAAGTATGGCCGTCCCCTATTGGGATGCACTATTAAACCTAAATTGGGGTTATCCGCTAAAAACTATGGTCGAGCAGTTTATGAATGTCTTCGCGGTGGATTATGCTCTTAACTCGACATCTTTTTCTCTG-TTAACTCGAACCTGG

TTTGTTGGGGTGTAATGGAATATGATGGAGCTCGAGTAGAAAGTATTGAGCTATTTATCAAGGGA-----------------AAGGGGTCTAGGGTTAGTGTCAATCAAAAGAATAAG

TTGGAACAACTTCGTAAGTTATCTTTGACAGAAAAATAG-AAAGGATC--AAAAAA

AATATAAAATTTTGAATCCCCCGGGACATTTTGATAAACCTTTGTT---AATTAATT

TGCTTTATATATATCGTGCGGAAATCCCTCGTTCATATGATTAGATTCTTTGATAGAAATAA-TAACAAAAAGGTATGTTGCTGCCATTTTTGAAAGGATTAAAAATCAAC

GAAGTAATGTCTAAACCCAATGATTAAAAAAAAAG-GATTTTAAAGGTTTCCGGA

ACAAGGAAAGACTCTTTTTAATTGTCGCAACAATTGATTGGGATCAATTCAAATCGATGTTAAATGAGACAAAACAAAGGGTATTTTAGACTGCTCAATAAATAA-----------TAAATGC-----TAAAGGATTTTGCGGGGGGGG--CTCCTTGAAACCGACCCAACTTG

AGTTATGAGTATACAAATGATTTTTT-----------TGAGGAAAGAA-----AAGAAAAGGC

TTAATTTTAATTCATTTGAGGATTGAGGATTTTATAGACTTTTTGATTGGTCATTCT

AATTTATACATACATTTT--TTTT-ATCATTTTT-CTCGAGCCGTACGAGGAGAAAAC

AAAACTTTCAAATTCAGAGAAACCCTGGAATTAAAAAA--AGGGCAATCCTGAGC

CAACTCC-ATTTTCTTTTTTTTTTT-CAAAAGGAAAATAAAGGATTAAGAAAGCAA

GAAAA--AGGGGATAGGTGCAGAGACTCAAAGGGAGCTGTTCTAAAAAATGGGGT

TGACTGTGTTATTATAAGTATAAGACTTCTTCCGTCTAAATTCCAAACCAA-----GA

ACAATAA-AAAGGGTGAAGAATATACGTACTGAAATTTTTAATGACAACCCGAAT

CTGTTCTGTATTTTTAATTT-TATTTTATTTTTTATATT-ATAGTAGAGATTTAGAAT

AGGGAATTTAAAATGCACGAATTGTTGTGAATCGATTCCAAGTTAAAAGCGGAAT

CCATATTTATT-CATCAAAACATTCACACTCACTCCATAATCTGATAGATCTTGTG

AAGAACTGATTAATCGGATGAGAATAAAGATAGAGTCCCGTTTTA-CATGTCAA-T

ACTGACAAC

>Bal4 AAGTGTTGGATTTAAAGCTGGTGTTAAAGATTACAAATTGACTTATTATACTCCTGAGTATGAAACCCTAGATACTGATATCTTGGCAGCATTCCGAGTAACTCCACAACCCGGAGTTCCACCCGAAGAAGCAGGGGCCGCAGTAGCCGCCGAATCTTCTACGGGTACATGGACAACTGTATGGACCGACGGACTTACCAGTCTTGATCGTTACAAAGGACGATGCTACCACATCGAGCCTGTTGCTGGAGAGGAAAATCAATATATTTGTTATGTAGCTTACCCCTTAGACCTTTTTGAGGAAGGCTCTGTTACTAACATGTTTACTTCCATTGTGGGTAATGTATTTGGGTTCAAAGCCTTGCGTGCTCTACGTTTGGAGGATTTGCGAATCCCTGTTGCTTATGTAAAAACTTTCCTAGGCCCGCCTCACGGTATCCAAGTTGAAAGAGATAAATTGAACAAGTATGGCCGTCCTCTATTGGGATGCACTATTAAACCTAAATTGGGGTTATCCGCTAAAAACTATGGTCGAGCAGTTTATGAATGTCTTCGCGGTGGATTATGCTCTTAACTCGACATCTTTTTATCTGTTTCACTCGAACCCGGTTTGTTGGGATGTAATGGAATATGATGGAGCTCGAGTAGAAAGTATTGAGTTATTTATCAAGGGA-----------------GAGGGGTCTAGGGTTAGTGTCAATCAAAAGAATCA

GTTGGAACAACTTCGTAAGTTATCTTTGACAGAAAAATAG-AAAGGAT-AAAAAA

AAAATATAAAATTTTGAATCCCCCGGGACATTTTGATAAACCTTTGTTGTTAATTA

ATTTGCTTTATATATATCGTGCGGAAATCCCTCGTTCATATGATTAGATTCTTTGATAGAAATAAATAACAAAAAGGTATGTTGCTGCTATTTTTGAAAGGATTAAAAATCAACGAAGTAATGTCTAAACCCAATGATTAAAAAAAAAAAGATTT-AAAGGCTTCC

GGAACAAGGAAAGACTCTTTTTAATTGTCGCAACAATTGATTGGGATCAATTCAAATCGATGTTAAATGAGACAAAACAAAGGGTATTTTAGACTGCTCAATAAATAATAAATAAATAATAAATGC-----TAAAGGATTTTGCGGGGGGGGG-CTCCTTGAAACCG

ACCCAACTTGAGTTATGAGTATACAAATGATTTTTT------------GAGGAAAGAA-----A

AGAAAAGGCTTAATTTTAATTCATTTGAGGATTGAGGATTTTATAGACTTTTTGAT

TGGTCATTCTAAGTTATACATACATTTT---TTTAATCGTTTTTTCTCGAGCCGTACG

AGGAGAAAACAAAACTTTCAAATTCAGAGAAACCCTGGAATTAAAAAA--AGGGC

AATCCTGAGCCAACTCCCATTTTCTTTTTTTTTT--CAAAAGGAAAATAAAGGATTA

AGAAAGCAAGAAAA--AGGGGATAGGTGCAGAGACTCAAAGGGAGCTGTTCTAAC

AAATGGGGTTGACTGTGTTATTATAAGTATAAGACTTCTTCCGTCTAAATTCCAAACCAA-----GAACAATAA-AAAGGGTGAAGAATATACGTACTGAAATTTTTAATGAC

AACCCGAATCTGTTCTGTATTTTTAATTT-TATTTTATTTTTTATATT-ATAGTAGAG

ATTTAGAATAGGGAATTAAAAATGCACGAATTGTTGTGAATCGATTCCAAGTTAAAAGCGGAATCCATATTTATT-CATCAAAACATTCACACTCACTCCATAATCTGATA

GATCTTGTGAAGAACTGATTAATCGGATGAGAATAAAGATAGAGTCCCGTTTTAG

CATGTCAA-TACTGATTAC

>Bal3 AAGTGTTGGATTTAAAGCTGGTGTTAAAGATTACAAATTGACTTATTATACTCCTGAGTATGAAACCCTAGATACTGATATCTTGGCAGCATTCCGAGTAACTCCACAACCCGGAGTTCCACCCGAAGAAGCAGGGGCCGCAGTAGCCGCCGAATCTTCTACGGGTACATGGACAACTGTATGGACCGACGGACTTACCAGTCTTGATCGTTACAAAGGACGATGCTACCACATCGAGCCTGTTGCTGGAGAGGAAAATCAATATATTTGTTATGTAGCTTACCCCTTAGACCTTTTTGAGGAAGGCTCTGTTACTAACATGTTTACTTCCATTGTGGGTAATGTATTTGGGTTCAAAGCCTTGCGTGCTCTACGTTTGGAGGATTTGCGAATCCCTGTTGCTTATGTAAAAACTTTCCTAGGCCCGCCTCACGGTATCCAAGTTGAAAGAGATAAATTGAACAAGTATGGCCGTCCTCTATTGGGATGCACTATTAAACCTAAATTGGGGTTATCCGCTAAAAACTATGGTCGAGCAGTTTATGAATGTCTTCGCGGTGGATTATGCTCTTAACTCGACATCTTTTTATCTGTTTCACTCGAACCCGGTTTGTTGGGATGTAATGGAATATGATGGAGCTCGAGTAGAAAGTATTGAGTTATTTATCAAGGGA-----------------GAGGGGTCTAGGGTTAGTGTCAATCAAAAGAATCA

GTTGGAACAACTTCGTAAGTTATCTTTGACAGAAAAATAG-AAAGGAT-AAAAAA

AAAATATAAAATTTTGAATCCCCCGGGACATTTTGATAAACCTTTGTTGTTAATTAATTTGCTTTATATATATCGTGCGGAAATCCCTCGTTCATATGATTAGATTCTTTGATAGAAATAAATAACAAAAAGGTATGTTGCTGCTATTTTTGAAAGGATTAAAAATCAACGAAGTAATGTCTAAACCCAATGATTAAAAAAAAAAAGATTT-AAAGGCTTCC

GGAACAAGGAAAGACTCTTTTTAATTGTCGCAACAATTGATTGGGATCAATTCAA

ATCGATGTTAAATGAGACAAAACAAAGGGTATTTTAGACTGCTCAATAAATAATAAATAAATAATAAATGC-----TAAAGGATTTTGCGGGGGGGGG-CTCCTTGAAACCG

ACCCAACTTGAGTTATGAGTATACAAATGATTTTTT------------GAGGAAAGAA-----A

AGAAAAGGCTTAATTTTAATTCATTTGAGGATTGAGGATTTTATAGACTTTTTGATTGGTCATTCTAAGTTATACATACATTTT---TTTAATCGTTTTTTCTCGAGCCGTACG

AGGAGAAAACAAAACTTTCAAATTCAGAGAAACCCTGAAATTAAAAAA--AGGGC

AATCCTGAGCCAACTCC-ATTTTCTTTTTTTTTTT-CAAAAGGAAAATAAAGGATTA

AGAAAGCAAGAAAA--AGGGGATAGGTGCAGAGACTCAAAGGGAGCTGTTCTAA

AAAATGGGGTTGACTGTGTTATTATAAGTATAAGACTTCTTCCGTCTAAATTCCAAACCAA-----GAACAATAA-AAAGGGTGAAGAATATACGTACTGAAATTTTTAATGA

CAACCCGAATCTGTTCTGTATTTTTAATTT-TATTTTATTTTTTATATT-ATAGTAGA

GATTTAGAATAGGGAATTTAAAATGCACGAATTGTTGTGAATCGATTCCAAGTTA

AAAGCGGAATCCATATTTATT-CATCAAAACATTCACACTCACTCCATAATCTGAT

AGATCTTGTGAAGAACTGATTAATCGGATGAGAATAAAGATAGAGTCCCGTTTTA

-CATGTCAA-TACTGACAAC

>Bal5 AAGTGTTGGATTTAAAGCTGGTGTTAAAGATTACAAATTGACTTATTATACTCCTGAGTATGAAACCCTAGATACTGATATCTTGGCAGCATTCCGAGTAACTCCACAACCCGGAGTTCCACCCGAAGAAGCAGGGGCCGCAGTAGCCGCCGAATCTTCTACGGGTACATGGACAACTGTATGGACCGACGGACTTACCAGTCTTGATCGTTACAAAGGACGATGCTACCACATCGAGCCTGTTGCTGGAGAGGAAAATCAATATATTTGTTATGTAGCTTACCCCTTAGACCTTTTTGAGGAAGGCTCTGTTACTAACATGTTTACTTCCATTGTGGGTAATGTATTTGGGTTCAAAGCCTTGCGTGCTCTACGTTTGGAGGATTTGCGAATCCCTGTTGCTTATGTAAAAACTTTCCTAGGCCCGCCTCACGGTATCCAAGTTGAAAGAGATAAATTGAACAAGTATGGCCGTCCTCTATTGGGATGCACTATTAAACCTAAATTGGGGTTATCCGCTAAAAACTATGGTCGAGCAGTTTATGAATGTCTTCGCGGTGGATTATGCTCTTAACTCGACATCTTTTTATCTGTTTCACTCGAACCCGGTTTGTTGGGATGTAATGGAATATGATGGAGCTCGAGTAGAAAGTATTGAGTTATTTATCAAGGGA-----------------GAGGGGTCTAGGGTTAGTGTCAATCAAAAGAATCA

GTTGGAACAACTTCGTAAGTTATCTTTGACAGAAAAATAG-AAAGGAT-AAAAAA

AAAATATAAAATTTTGAATCCCCCGGGACATTTTGATAAACCTTTGTTGTTAATTA

ATTTGCTTTATATATATCGTGCGGAAATCCCTCGTTCATATGATTAGATTCTTTGATAGAAATAAATAACAAAAAGGTATGTTGCTGCTATTTTTGAAAGGATTAAAAATC

AACGAAGTAATGTCTAAACCCAATGATTAAAAAAAAAAAGATTT-AAAGGCTTCC

GGAACAAGGAAAGACTCTTTTTAATTGTCGCAACAATTGATTGGGATCAATTCAA

ATCGATGTTAAATGAGACAAAACAAAGGGTATTTTAGACTGCTCAATACATAATAAATAAATAATAAATGC-----TAAAGGATTTTGCGGGGGGGGGGCTCCTTGAAACCG

ACCCAACTTGAGTTATGAGTATACAAATGATTTTTT------------GAGGAAAGAA-----A

AGAAAAGGCTTAATTTTAATTCATTTGAGGATTGAGGATTTTATAGACTTTTTGAT

TGGTCATTCTAAGTTATACATACATTTT---TTTAATCGTTTTTTCTCGAGCCGTACG

AGGAGAAAACAAAACTTTCAAATTCAGAGAAACCCTGGAATTAAAAAA--AGGGC

AATCCTGAGCCAACTCC-ATTTT-------------CAAAAGGAAAATAAAGGATTAAGAAA

GCAAGAAAAA-AAGGGATAGGTGCAGAGACTCAAAGGGAGCTGTTCTAACAAAT

GGGGTTGACTGTGTTATTATAAGTATAAGACTTCTTCCGTCGAAATTCCAAACCAA-----GAACAATAATAAAGGGTGAAGAATATACGTACTGAAATTTTTAATGACAAC

CCGAATCTGTTCTGTATTTTTAATTT-TATTTTATTTTAAATATT-ATAGTAGAGATT

TAGAATAGGGAATTTAAAATGCACGAATTGTTGTGAATCGATTCCAAGTTAAAAG

CGGAATCCATATTTATT-CATCAAAACATTCACACTCACTCCATAATCTGATAGAT

CTTGTGAAGAACTGATTAATCGGATGAGAATAAAGATAGAGTCCCGTTTTA-CAT

GTCAA-TACTGATTAC

>Ari AAGTGTTGGATTTAAAGCTGGTGTTAAAGATTACAAATTGACTTATTATACTCCTGAGTATGAAACCCTAGATACTGATATCTTGGCAGCATTCCGAGTAACTCCACAACCCGGAGTTCCACCCGAAGAAGCAGGGGCCGCAGTAGCCGCCGAATCTTCTACGGGTACATGGACAACTGTATGGACCGACGGACTTACCAGTCTTGATCGTTACAAAGGACGATGCTACCACATCGAGCCTGTTGCTGGAGAGGAAAATCAATATATTTGTTATGTAGCTTACCCCTTAGACCTTTTTGAGGAAGGCTCTGTTACTAACATGTTTACTTCCATTGTGGGTAATGTATTTGGGTTCAAAGCCTTGCGTGCTCTACGTTTGGAGGATTTGCGAATCCCTGTTGCTTATGTAAAAACTTTCCTAGGCCCGCCTCACGGTATCCAAGTTGAAAGAGATAAATTGAACAAGTATGGCCGTCCTCTATTGGGATGCACTATTAAACCTAAATTGGGGTTATCCGCTAAAAACTATGGTCGAGCAGTTTATGAATGTCTTCGCGGTGGATTATGCTCTTAACTCGACATCTTTTTATCTGTTTCACTCGAACCCTGTTTGTTGGGATGTAATGGAATATGATGGAGCTCGAGTAGAAAGTATTGAGTTATTTATCAAGGGA-----------------GAGGGGTCTAGGGTTAGTGTCAATCAAAAGAATCA

GTTGGAACAACTTCGTAAGTTATCTTTGACAGAAAAATAG-AAAGGATC-AAAAA

AAAATATAAAATTTTGAATCCCCCGGGACATTTTGATAAACCTTTGTTGTTAATTA

ATTTGCTTTATATATATCGTGCGGAAATCCCTCGTTCATATGATTAAATTCTTTGATAGAAATAAATAACAAAAAGGTATGTTGCTGCTATTTTTGAAAGGATTAAAAATC

AACGAAGTAATGTCTAAACCCAATGATTAAAAAAAAAAAGATTT-AAAGGCTTCC

GGAACAAGGAAAGACTCTTTTTAATTGTCGCAACAATTGATTGGGATCAATTCAAATCGATGTTAAATGAGACAAAACAAAGGGTATTTTAGACTGCTCAATAAATAA-----------TAAATGC-----TAAAGGATTTTGCGGGGGGGGG-CTCCTTGAAACCGACCCAA

CTTGAGTTATGAGTATACAAATGATTTTTT------------GAGGAAAGAA-----AAGAAA

AGGCTTAATTTTAATTCATTTGAGGATTGAGGATTTTATAGACTTTTTGATTGGTC

ATTCTAAGTTATACATACATTTT---TTTAATCGTTTTTTCTCGAGCCGTACGAGGAG

AAAACAAAACTTTCAAATTCAGAGAAACCCTGGAATTAAAAAA--AGGGCAATCC

TGAGCCAACTCC-ATTTT-------------CAAAAGGAAAATAAAGGATTAAGAAAGCAA

GAAAAA-AAGGGATAGGTGCAGAGACTCAAAGGGAGCTGTTCTAACAAATGGGG

TTGACTGTGTTATTATAAGTATAAGACTTCTTCCGTCGAAATTCCAAACCAA-----G

AACAATAATAAAGGGTGAAGAATATCCGTACTGAAATTTTTAATGACAACCCGAATCTGTTCTGTATTTTTAATTT-TATTTTATTTTAAATATT-ATAGTAGAGATTTAGA

ATAGGGAATTTAAAATGCACGAATTGTTGTGAATCGATTCCAAGTTAAAAGCGGA

ATCCATATTTATT-CATCAAAACATTCACACTCACTCCATAATCTGATAGATCTTGT

GAAGAACTGATTAATCGGATGAGAATAAAGATAGAGTCCCGTTTTA-CATGTCAA-TACTGATTAC

>Bal6 AAGTGTTGGATTTAAAGCTGGTGTTAAAGATTACAAATTGACTTATTATACTCCTGAGTATGAAACCCTAGATACTGATATCTTGGCAGCATTCCGAGTAACTCCACAACCCGGAGTTCCACCCGAAGAAGCAGGGGCCGCAGTAGCCGCCGAATCTTCTACGGGTACATGGACAACTGTATGGACCGACGGACTTACCAGTCTTGATCGTTACAAAGGACGATGCTACCACATCGAGCCTGTTGCTGGAGAGGAAAATCAATATATTTGTTATGTAGCTTACCCCTTAGACCTTTTTGAGGAAGGCTCTGTTACTAACATGTTTACTTCCATTGTGGGTAATGTATTTGGGTTCAAAGCCTTGCGTGCTCTACGTTTGGAGGATTTGCGAATCCCTGTTGCTTATGTAAAAACTTTCCTAGGCCCGCCTCACGGTATCCAAGTTGAAAGAGATAAATTGAACAAGTATGGCCGTCCTCTATTGGGATGCACTATTAAACCTAAATTGGGGTTATCCGCTAAAAACTATGGTCGAGCAGTTTATGAATGTCTTCGCGGTGGATTATGCTCTTAACTCGACATCTTTTTATCTGTTTCACTCGAACCCGGTTTGTTGGGATGTAATGGAATATGATGGAGCTCGAGTAGAAAGTATTGAGTTATTTATCAAGGGA-----------------GAGGGGTCTAGGGTTAGTGTCAATCAAAAGAATCA

GTTGGAACAACTTCGTAAGTTATCTTTGACAGAAAAATAG-AAAGGAT-AAAAAA

AAAATATAAAATTTTGAATCCCCCGGGACATTTTGATAAACCTTTGTTGTTAATTAATTTGCTTTATATATATCGTGCGGAAATCCCTCGTTCATATGATTAGATTCTTTGATAGAAATAAATAACAAAAAGGTATGTTGCTGCTATTTTTGAAAGGATTAAAAATCAACGAAGTAATGTCTAAACCCAATGATTAAAAAAAAAAAGATTT-AAAGGCTTCC

GGAACAAGGAAAGACTCTTTTTAATTGTCGCAACAATTGATTGGGATCAATTCAA

ATCGATGTTAAATGAGACAAAACAAAGGGTATTTTAGACTGCTCAATAAATAATAAATAAATAATAAATGC-----TAAAGGATTTTGCGGGGGGGGG-CTCCTTGAAACCG

ACCCAACTTGAGTTATGAGTATACAAATGATTTTTT------------GAGGAAAGAA-----A

AGAAAAGGCTTAATTTTAATTCATTTGAGGATTGAGGATTTTATAGACTTTTTGAT

TGGTCATTCTAAGTTATACATACATTTT---TTTAATCGTTTTTTCTCGAGCCGTACG

AGGAGAAAACAAAACTTTCAAATTCAGAGAAACCCTGGAATTAAAAAA--AGGGC

AATCCTGAGCCAACTCCCATTTTCTTTTTTTTTT--CAAAAGGAAAATAAAGGATTA

AGAAAGCAAGAAAAA-AAGGGATAGGTGCAGAGACTCAAAGGGAGCTGTTCTAA

CAAATGGGGTTGACTGTGTTATTATAAGTATAAGACTTCTTCCGTCTAAATTCCAAACCAA-----GAACAATAATAAAGGGTGAAGAATATACGTACTGAAATTTTTAATGA

CAACCCGAATCTGTTCTGTATTTTTAATTT-TATTTTATTTTTTATATT-ATAGTAGA

GATTTAGAATAGGGAATTTAAAATGCACGAATTGTTGTGAATCGATTCCAAGTTA

AAAGCGGAATCCATATTTATT-CATCAAAACATTCACACTCACTCCATAATCTGAT

AGATCTTGTGAAGAACTGATTAATCGGATGAGAATAAAGATAGAGTCCCGTTTTAGCATGTCAA-TACTGATTAC

>Cen1 AAGTGTTGGATTTAAAGCTGGTGTTAAAGATTACAAATTGACTTATTATACTCCTGAGTATGAAACCCTAGATACTGATATCTTGGCAGCATTCCGAGTAACTCCACAACCCGGAGTTCCACCCGAAGAAGCAGGGGCCGCAGTAGCCGCCGAATCTTCTACGGGTACATGGACAACTGTATGGACCGACGGACTTACCAGTCTTGATCGTTACAAAGGACGATGCTACCACATCGAGCCTGTTGCTGGAGAGGAAAATCAATATATTTGTTATGTAGCTTACCCCTTAGACCTTTTTGAGGAAGGCTCTGTTACTAACATGTTTACTTCCATTGTGGGTAATGTATTTGGGTTCAAAGCCTTGCGTGCTCTACGTTTGGAGGATTTGCGAATCCCTGTTGCTTATGTAAAAACGTTCCTAGGCCCGCCTCACGGTATCCAAGTTGAAAGAGATAAATTGAACAAGTATGGCCGTCCCCTATTGGGATGCACTATTAAACCTAAATTGGGGTTATCCGCTAAAAACTATGGTCGAGCAGTTTATGAATGTCTTCGCGGTGGATTATGCTCTTAACTCGACATCTTTTTCTCTGTTTAACTCGAACCCGGTTTGTTGGGGTGTAATGGAATATGATGGAGCTCGAGTAGAAAGTATTGAGCTATTTATCAAGGGA-----------------AAGGGGTCTAGGGTTAGTGTCAATCAAAAGAATAA

GTTGGAACAACTTCGTAAGTTATCTTTGACAGAAAAATAG-AAAGGATCAAAAAA

AAAAGATAAAATTTTGAATCCCCCGGGACATTTTGATAAACCTTTGTC---AATTAA

TTTGCTTTATATATATCGTGCGGAAATCCCTCGTTCATATGATTAGATTCTTTGATAGAAATAA-TAACAAAAAGGTATGTTGCTGCCATTTTTGAAAGGATTAAAAATCA

ACGAAGTAATGTCTAAACCCAATGATTAAAAAAAAG--GATTT-AAAGGCTTCCGG

AACAAGGAAAGACTCTTTTTAATTGTCGCAACAATTGATTGGGATCAATTCAAATCGATGTTAAATGAGACAAAACAAAGGGTATTTTAGACTGCTCAATAAATAA-----------TAAATGC-----TAAAGGATTTTGCGGGGGGGG--CTCCTTGAAACCGACCCAACTT

GAGTTATGAGTATACAAATGATTTTTT-----------TGAGGAAAGAA-----AAGAAAAGG

CTTAATTTTAATTCATTTGAGGATTGAGGATTTTATAGACTTTTTGATTGGTCATTC

TAATTTATACATACATTTT--TTTTAATCATTTTT-CTCGAGCCGTACGAGGAGAAA

ACAAAACTTTCAAATTCAGAGAAACCCTGGAATTAAAAAA--AGGGCAATCCTGA

GCCAACTCCCATTTTCTTTTTTTTTT--CAAAAGGAAAATAAAGGATTAAGAAAGC

AAGAAAA--AGGGGATAGGTGCAGAGACTCAAAGGGAGCTGTTCTAACAAATGGG

GTTGACTGTGTTATTATAAGTATAAGACTTCTTCCGTCTAAATTCCAAACCAA-----GAACAATAA-AAAGGGTGAAGAATATACGTACTGAAATTTTTAATGACAACCCGA

ATCTGTTCTGTATTTTTAATTT-TATTTTATTTATTATATT-ATAGTAGAGATTTAGA

ATAGGGAATTAAAAATGCACGAATTGTTGTGAATCGATTCCAAGTTAAAAGCGG

AATCCATATTTATT-CATCAAAACATTCACACTCACTCCATAATCTGATAGATCTT

GTGAAGAACTGATTAATCGGATGAGAATAAAGATAGAGTCCCGTTTTAGCATGTCAA-TACTGATTAC

>Ape2 AAGTGTTGGATTTAAAGCTGGTGTTAAAGATTACAAATTGACTTATTATACTCCTGAGTATGAAACCCTAGATACTGATATCTTAGCAGCATTCCGAGTAACTCCACAACCCGGAGTTCCACCCGAAGAAGCAGGGGCCGCAGTAGCCGCCGAATCTTCTACGGGTACATGGACAACTGTATGGACCGACGGACTTACCAGTCTTGATCGTTACAAAGGACGATGCTACCACATCGAGCCTGTTGCTGGAGAGGAAAATCAATATATTTGTTATGTAGCTTACCCCTTAGACCTTTTTGAGGAAGGCTCTGTTACTAACATGTTTACTTCCATTGTGGGTAATGTATTTGGGTTCAAAGCCTTGCGTGCTCTACGTTTGGAGGATTTGCGAATCCCTGTTGCTTATGTAAAAACTTTCCTAGGCCCGCCTCACGGTATCCAAGTTGAAAGAGATAAATTGAACAAGTATGGCCGTCCTCTATTGGGATGCACTATTAAACCTAAATTGGGGTTATCCGCTAAAAACTATGGTCGAGCAGTTTATGAATGTCTTCGCGGTGGATTATGCTCTTAACTCGACATCTTTTTATCTGTTTCACTCGAACCCGGTTTGTTGGGATGTAATGGAATATGATGGAGCTCGAGTAGAAAGTATTGAGTTATTTATCAAGGGA-----------------GAGGGGTCTAGGGTTAGTGTCAATCAAAAGAATCA

GTTGGAACAACTTCGTAAGTTATCTTTGACAGAAAAATAG-AAAGGATC-AAAAA

AAAATATAAAATTTTGAATCCCCCGGGACATTTTGATAAACCTTTGTT---AATTAA

TTTGCTTTATATATATCGTGCGGAAATCCCTCGTTCATATGATTAGATTCTTTGATAGAAATAAATAACAAAAAGGTATGTTGCTGCTATTTTTGAAAGGATTAAAAATCAACGAAGTAATGTCTAAACCCAATGATTAAAAAAAAA--GATTT-AAAGGCTTCCGG

AACAAGGAAAGACTCTTTTTAATTGTCGCAACAATTGATTGGGATCAATTCAAATCGCTGTTAAATGAGACAAAACAAAGGGTATTTTAGACTGCTCAATAAATAA-----------TAAATGC-----TAAAGGATTTTGCGGGGGGGGGGCTCCTTGAAACCGACCCAACT

TGAGTTATGAGTATACAAATGATTTTTT------------GAGGAAAGAA-----AAGAAAAG

GCTTAATTTTAATTCATTTGAGGATTGAGGATTTTATAGACTTTTTGATTGGTCATTCTAATTTATACATACATTTT---TTTAATCGTTTTTTCTCGAGCCGTACGAGGAGAA

AACAAAACTTTCAAATTCAGAGAAACCCTGGAATTAAAAAA--AGGGCAATCCTG

AGCCAACTCCCATTTTCTTTTTTTTTTTTCAAAAGGAAAATAAAGGATTAAGAAAGCAAGAAAA--AGGGGATAGGTGCAGAGACTCAAAGGGAGCTGTTCTAACAAATGG

GGTTGACTGTGTTATTATAAGTATAAGACTTCTTCCGTCTAAATTCCAAACCAA-----GAACAATAA-AAAGGGTGAAGAATATACGTACTGAAATTTTTAATGACAACCCG

AATCTGTTCTGTATTTTTAATTT-TATTTTATTTTTTATATT-ATAGTAGAGATTTAG

AATAGGGAATTAAAAATGCACGAATTGTTGTGAATCGATTCCAAGTTAAAAGCG

GAATCCATATTTATT-CATCAAAACATTCACACTCACTCCATAATCTGATAGATCT

TGTGAAGAACTGATTAATCGGATGAGAATAAAGATAGAGTCCCGTTTTAGCATGTCAA-TACTGATTAC

>Ape1 AAGTGTTGGATTTAAAGCTGGTGTTAAAGATTACAAATTGACTTATTATACTCCTGAGTATGAAACCCTAGATACTGATATCTTAGCAGCATTCCGAGTAACTCCACAACCCGGAGTTCCACCCGAAGAAGCAGGGGCCGCAGTAGCCGCCGAATCTTCTACGGGTACATGGACAACTGTATGGACCGACGGACTTACCAGTCTTGATCGTTACAAAGGACGATGCTACCACATCGAGCCTGTTGCTGGAGAGGAAAATCAATATATTTGTTATGTAGCTTACCCCTTAGACCTTTTTGAGGAAGGCTCTGTTACTAACATGTTTACTTCCATTGTGGGTAATGTATTTGGGTTCAAAGCCTTGCGTGCTCTACGTTTGGAGGATTTGCGAATCCCTGTTGCTTATGTAAAAACTTTCCTAGGCCCGCCTCACGGTATCCAAGTTGAAAGAGATAAATTGAACAAGTATGGCCGTCCTCTATTGGGATGCACTATTAAACCTAAATTGGGGTTATCCGCTAAAAACTATGGTCGAGCAGTTTATGAATGTCTTCGCGGTGGATTATGCTCTTAACTCGACATCTTTTTATCTGTTTCACTCGAACCCGGTTTGTTGGGATGTAATGGAATATGATGGAGCTCGAGTAGAAAGTATTGAGTTATTTATCAAGGGA-----------------GAGGGGTCTAGGGTTAGTGTCAATCAAAAGAATCA

GTTGGAACAACTTCGTAAGTTATCTTTGACAGAAAAATAG-AAAGGATC-AAAAA

AAAATATAAAATTTTGAATCCCCCGGGACATTTTGATAAACCTTTGTT---AATTAA

TTTGCTTTATATATATCGTGCGGAAATCCCTCGTTCATATGATTAGATTCTTTGATAGAAATAAATAACAAAAAGGTATGTTGCTGCTATTTTTGAAAGGATTAAAAATCAACGAAGTAATGTCTAAACCCAATGATTAAAAAAAAA--GATTT-AAAGGCTTCCGG

AACAAGGAAAGACTCTTTTTAATTGTCGCAACAATTGATTGGGATCAATTCAAATCGCTGTTAAATGAGACAAAACAAAGGGTATTTTAGACTGCTCAATAAATAA-----------TAAATGC-----TAAAGGATTTTGCGGGGGGGGGGCTCCTTGAAACCGACCCAACT

TGAGTTATGAGTATACAAATGATTTTTT------------GAGGAAAGAA-----AAGAAAAG

GCTTAATTTTAATTCATTTGAGGATTGAGGATTTTATAGACTTTTTGATTGGTCATTCTAATTTATACATACATTTT---TTTAATCGTTTTTTCTCGAGCCGTACGAGGAGAA

AACAAAACTTTCAAATTCAGAGAAACCCTGGAATTAAAAAA--AGGGCAATCCTG

AGCCAACTCCCATTTTCTTTTTTTTTT--CAAAAGGAAAATAAAGGATTAAGAAAG

CAAGAAAA--AGGGGATAGGTGCAGAGACTCAAAGGGAGCTGTTCTAACAAATGG

GGTTGACTGTGTTATTATAAGTATAAGACTTCTTCCGTCTAAATTCCAAACCAA-----GAACAATAA-AAAGGGTGAAGAATATACGTACTGAAATTTTTAATGACAACCCG

AATCTGTTCTGTATTTTTAATTT-TATTTTATTTTTTATATT-ATAGTAGAGATTTAG

AATAGGGAATTAAAAATGCACGAATTGTTGTGAATCGATTCCAAGTTAAAAGCG

GAATCCATATTTATT-CATCAAAACATTCACACTCACTCCATAATCTGATAGATCT

TGTGAAGAACTGATTAATCGGATGAGAATAAAGATAGAGTCCCGTTTTAGCATGTCAA-TACTGATTAC

>Ape3 AAGTGTTGGATTTAAAGCTGGTGTTAAAGATTACAAATTGACTTATTATACTCCTGAGTATGAAACCCTAGATACTGATATCTTGGCAGCATTCCGAGTAACTCCACAACCCGGAGTTCCACCCGAAGAAGCAGGGGCCGCAGTAGCCGCCGAATCTTCTACGGGTACATGGACAACTGTATGGACCGACGGACTTACCAGTCTTGATCGTTACAAAGGACGATGCTACCACATCGAGCCTGTTGCTGGAGAGGAAAATCAATATATTTGTTATGTAGCTTACCCCTTAGACCTTTTTGAGGAAGGCTCTGTTACTAACATGTTTACTTCCATTGTGGGTAATGTATTTGGGTTCAAAGCCTTGCGTGCTCTACGTTTGGAGGATTTGCGAATCCCTGTTGCTTATGTAAAAACTTTCCTAGGCCCGCCTCACGGTATCCAAGTTGAAAGAGATAAATTGAACAAGTATGGCCGTCCTCTATTGGGATGCACTATTAAACCTAAATTGGGGTTATCCGCTAAAAACTATGGTCGAGCAGTTTATGAATGTCTTCGCGGTGGATTATGCTCTTAACTCGACATCTTTTTATCTGTTTCACTCGAACCCGGTTTGTTGGGATGTAATGGAATATGATGGAGCTCGAGTAGAAAGTATTGAGTTATTTATCAAGGGA-----------------GAGGGGTCTAGGGTTAGTGTCAATCAAAAGAATCA

GTTGGAACAACTTCGTAAGTTATCTTTGACAGAAAAATAG-AAAGGAT-AAAAAA

AAAATATAAAATTTTGAATCCCCCGGGACATTTTGATAAACCTTTGTTGTTAATTA

ATTTGCTTTATATATATCGTGCGGAAATCCCTCGTTCATATGATTAGATTCTTTGATAGAAATAAATAACAAAAAGGTATGTTGCTGCTATTTTTGAAAGGATTAAAAATCAACGAAGTAATGTCTAAACCCAATGATTAAAAAAAAAAAGATTT-AAAGGCTTCC

GGAACAAGGAAAGACTCTTTTTAATTGTCGCAACAATTGATTGGGATCAATTCAAATCGATGTTAAATGAGACAAAACAAAGGGTATTTTAGACTGCTCAATAAATAATAAATAAATAATAAATGC-----TAAAGGATTTTGCGGGGGGGGG-CTCCTTGAAACCG

ACCCAACTTGAGTTATGAGTATACAAATGATTTTTT------------GAGGAAAGAA-----A

AGAAAAGGCTTAATTTTAATTCATTTGAGGATTGAGGATTTTATAGACTTTTTGATTGGTCATTCTAAGTTATACATACATTTT---TTTAATCGTTTTTTCTCGAGCCGTACG

AGGAGAAAACAAAACTTTCAAATTCAGAGAAACCCTGGAATTAAAAAA--AGGGC

AATCCTGAGCCAACTCCCATTTTCTTTTTTTTTT--CAAAAGGAAAATAAAGGATTA

AGAAAGCAAGAAAA--AGGGGATAGGTGCAGAGACTCAAAGGGAGCTGTTCTAAC

AAATGGGGTTGACTGTGTTATTATAAGTATAAGACTTCTTCCGTCTAAATTCCAAACCAA-----GAACAATAA-AAAGGGTGAAGAATATACGTACTGAAATTTTTAATGAC

AACCCGAATCTGTTCTGTATTTTTAATTT-TATTTTATTTTTTATATT-ATAGTAGAG

ATTTAGAATAGGGAATTAAAAATGCACGAATTGCTGTGAATCGATTCCAAGTTAA

AAGCGGAATCCATATTTATT-CATCAAAACATTCACACTCACTCCATAATCTGATA

GATCTTGTGAAGAACTGATTAATCGGATGAGAATAAAGATAGAGTCCCGTTTTAG

CATGTCAA-TACTGATTAC

>Din AAGTGTTGGATTTAAAGCTGGTGTTAAAGATTACAAATTGACTTATTATACTCCTGAGTATGAAACCCTAGATACTGATATCTTGGCAGCATTCCGAGTAACTCCACAACCCGGAGTTCCACCCGAAGAAGCAGGGGCCGCAGTAGCCGCCGAATCTTCTACGGGTACATGGACAACTGTATGGACCGACGGACTTACCAGTCTTGATCGTTACAAAGGACGATGCTACCACATCGAGCCTGTTGCTGGAGAGGAAAATCAATATATTTGTTATGTAGCTTACCCCTTAGACCTTTTTGAGGAAGGCTCTGTTACTAACATGTTTACTTCCATTGTGGGTAATGTATTTGGGTTCAAAGCCTTGCGTGCTCTACGTTTGGAGGATTTGCGAATCCCTGTTGCTTATGTAAAAACTTTCCTAGGCCCGCCTCACGGTATCCAAGTTGAAAGAGATAAATTGAACAAGTATGGCCGTCCTCTATTGGGATGCACTATTAAACCTAAATTGGGGTTATCCGCTAAAAACTATGGTCGAGCAGTTTATGAATGTCTTCGCGGTGGATTGTGCTCTTAACTCGACATCTTTTTCTCTG-TTAACTCGAACCCGG

TTTGTTGGGGTGTAATGGAATATGATGGAGCTCGAGTAGAAAGTATTGAGCTATTTATCAAGGGA-----------------AAGGGGTCTAGGGTTAGTGTCAATCAAAAGAATAAG

TTGGAACAACTTCGTAAGTTATCTTTGACAGAAAAATAG-AAAGGATC-AAAAAA

AAATATAAAATTTTGAATCCCCCGGGACATTTTGATAAACCTTTGTT---AATTAAT

TTGCTTTATATATATCGTGCGGAAATCCCTTGTTCATATGATTAGATTCTTTGATAGAAATAAATAACAAAAAGGTATGTTGCTGCCATTTTTGAAAGGATTAAAAATCAACGAAGTAATGTCTAAACCCAATGATTAAAAAAAAAAGGATTT-AAAGGCTTCCGG

AACAAGGAAAGACTCTTTTTAATTGTCGCAACAATTGATTGGGATCAATTCAAATCGATGTTAAATGAGACAAAACAAAGGGTATTTTAGACTGCTCAATAAATAA-----------TAAATGC-----TAAAGGATTTTGCGGGGGGGGG-CTCCTTGAAACCGACCCAACT

TGAGTTATGAGTATACAAATGATTTTTT-----------TGAGGAAAGAA-----AAGAAAAG

GCTTAATTTTAATTCATTTGAGGATTGAGGATTTTATAGACTTTTTGATTGGTCATTCTAATTTATACATACATTTT---TTTAATCATTTTT-CTCGAGCCGTACGAGGAGAA

AACAAAACTTTCAAATTCAGAGAAACCCTGGAATTAAAAAA--AGGGCAATCCTG

AGCCAACTCCCATTTTCTTTTTTTTTTTTCAAAAGGAAAATAAAGGATTAAGAAAGCAAGAAAA--AGGGGATAGGTGCAGAGACTCAAAGGGAGCTGTTCTAACAAATGG

GGTTGACTGTGTTATTATAAGTATAAGACTTCTTCCGTCTAAATTCCAAACCAA-----GAACAATAA-AAAGGGTGAAGAATATACGTACTGAAATTTTTAATGACAACCCG

AATCTGTTCTGTATTTTTAATTT-TATTTTATTTTTTATATT-ATAGTAGAGATTTAG

AATAGGGAATTAAAAATGCACGAATTGCTGTGAATCGATTCCAAGTTAAAAGCGGAATCCATATTTATT-CATCAAAACATTCACACTCACTCCATAATCTGATAGATCT

TGTGAAGAACTGATTAATCGGATGAGAATAAAGATAGAGTCCCGTTTTAGCATGT

CAA-TACTGATTAC

>Pyr4 AAGTGTTGGATTTAAAGCTGGTGTTAAAGATTACAAATTGACTTATTATACCCCTGAGTATGAAACCCTAGATACTGATATCTTGGCAGCATTCCGAGTAACTCCACAACCCGGAGTTCCACCCGAAGAAGCAGGGGCCGCAGTAGCCGCCGAATCTTCTACGGGTACATGGACAACTGTATGGACCGACGGACTTACCAGTCTTGATCGTTACAAAGGACGATGCTACCACATCGAGCCTGTTGCTGGAGAGGAAAATCAATATATTTGTTATGTAGCTTACCCCTTAGACCTTTTTGAGGAAGGCTCTGTTACTAACATGTTTACTTCCATTGTGGGTAATGTATTTGGGTTCAAAGCCTTGCGTGCTCTACGTTTGGAGGATTTGCGAATCCCTGTTGCTTATGTAAAAACTTTCCTAGGCCCGCCTCACGGTATCCAAGTTGAAAGAGATAAATTGAACAAGTATGGCCGTCCCCTATTGGGATGCACTATTAAACCTAAATTGGGGTTATCCGCTAAAAACTATGGTCGAGCAGTTTATGAATGTCTTCGCGGTGGATTATGCTCTTAACTCGACATCTTTTTATCTGTTTCACTCGAACCCGGTTTGTTGGGATGTAATGGAATATGATGGAGCTCGAGTAGAAAGTATTGAGTTATTTATCAAGGGA-----------------GAGGGGTCTAGGGTTAGTGTCAATCAAAAGAATCA

GTTGGAACAACTTCGTAAGTTATCTTTGACAGAAAAATAG-AAAGGAT-AAAAAA

AAAATATAAAATTTTGAATCCCCCGGGACATTTTGATAAACCTTTGTTGTTAATTA

ATTTGCTTTATATATATCGTGCGGAAATCCCTCGTTCATATGATTAGATTCTTTGATAGAAATAAATAACAAAAAGGTATGTTGCTGCTATTTTTGAAAGGATTAAAAATCAACGAAGTAATGTCTAAACCCAATGATTAAAAAAAAAAAGATTT-AAAGGCTTCC

GGAACAAGGAAAGACTCTTTTTAATTGTCGCAACAATTGATTGGGATCAATTCAA

ATCGATGTTAAATGAGACAAAACAAAGGGTATTTTAGACTGCTCAATAAATAA-----------TAAATGC-----TAAAGGATTTTGCGGGGGGGGG-CTCCTTGAAACCGACCCAA

CTTGAGTTATGAGTATACAAATGATTTTTT------------GAGGAAAGAA-----AAGAAA

AGGCTTAATTTTAATTCATTTGAGGATTGAGGATTTTATAGACTTTTTGATTGGTC

ATTCTAATTTATACATACATTTT---TTTAATCGTTTTTTCTCGAGCCGTACGAGGAG

AAAACAAAACTTTCAAATTCAGAGAAACCCTGGAATTAAAAAA--AGGGCAATCC

TGAGCCAACTCC-ATTTTCCTTTTTTT----CAAAAGCAAAAGAAAGGATGAAGAAA

GCAAGAAAAA-AAGGGATAGGTGCAGAGACTCAAAGGGAGCTGTTCTAACAAAT

GGGGTTGACTGTGTTGTTATAAGTATAAGACTTCTTCCGTCTAAATTCCAAACCAA-----GAA-------AAAGGGTGAAGAATATACGTACTGAAATGATTAATGACAACCCGA

AAATGTTTTTTATTTTCTATTT-TAATAA----------TT-ATAGTAGAGATTTAGAATAG

GAAATTAAAAATGCAAGAATTGTTGTGAATTGATTCCAAGTTAAAAGCGGAATCCATATTTATT-CATGAAAACATTCACACTCACTCCATAGTCTGATAGATCTTGTGAA

GAACTGATTAATCAGATGAGAATAAAGATAGAGTCCCGTTTTAGCATGTCAA-TACTGACAAC
