# Supplementary material for: A glacial survivor of the alpine Mediterranean region: phylogenetic and phylogeographic insights into Silene ciliata Pourr. (Caryophyllaceae)
Source: PeerJ. 2015 Aug 20;3:e1193. doi: 10.7717/peerj.1193 (PMC4548490; doi:10.7717/peerj.1193)
Supplement: Table S1 — DNA samples used for the study. The table shows the acronym given for each Silene ciliata population gathered (“Name”), the “Country” where these populations were collected and the topographic details of the collection sites (Location, Altitude, MGRS coordinates). There is also a reference to the herbarium code or specimen collection where the material is deposited (“Collection”). Footnote: Individuals from Rey Juan Carlos Database are managed by Alfredo García-Fernández (alfredo.garcia@urjc.es), Jose María Iriondo (jose.iriondo@urjc.es) or URJC Germplasm bank (patricia.alonso@urjc.es). [file peerj-03-1193-s005.docx]

| Name | Country | Location | Altitude(m) | MGRS | Collection |
| --- | --- | --- | --- | --- | --- |
| Can1 | ES | Cantabrian Range. Valley of San Emiliano. Around Puerto de la Ventana, east slope. | 1642 | 29TQH4477 | Rey Juan Carlos Database Sci-001-LG |
| Can2 | ES | Cantabrian Range. Pico Jario. Picos de Europa. Western massif. | 1900 | 30TUN3712 | Rey Juan Carlos Database Sci-002-LG |
| Can3 | ES | Cantabrian Range. Jou Sin Tierre. Picos de Europa. Spain. | 1881 | 30TUN5150 | Rey Juan Carlos Database Sci-001-AB |
| Ibe1 | ES | Iberian System. Laguna de los Patos. Neila. Burgos. | 1900 | 30TVM9646 | Rey Juan Carlos Database Sci-001-AL |
| Ibe2 | ES | Iberian System. Sierra de la Demanda. Pico de San Lorenzo. | 2278 | 30TWM0276 | Rey Juan Carlos Database Sci-003-LG |
| Pyr1 | ES | Pyrenees mountain range. Yésero. Ascending towards Puerto de Otal. | 1931 | 30TYN2920 | Rey Juan Carlos Database Sci-004-LG |
| Pyr2 | ES | Pyrenees mountain range. P.N.Ordesa; Solana de la Carriata. Torla, Huesca. | 1350-1780 | 30TYN4026 | Herbarium JACA. Instituto Pirenaico de Ecología. R207074 |
| Pyr3 | ES | Pyrenees mountain range. Sierra de Querol, north of Puig de les Morreres. | 2100-2200 | 31T CG7967 | Herbarium of Faculty of Farmacology ,University of Barcelona. BCN96966 |
| Pyr5 | ES | Pyrenees mountain range. Ripolles, Toses, north of Collet de San Salvador. | 2161 | 31TDG1980 | Herbarium of Faculty of Farmacology, University of Barcelona. BCN95945 |
| Cen2 | ES | Central System. Pico del Aguila. Sierra de Bejar. | 1950 | 30TTK7079 | Real Jardín Botanico de Madrid. CSIC MA880762 |
| Cen3 | ES | Central System. Sierra de Guadarrama. Pico Cabeza de Hierro. Between C.H. Mayor and C.H. Menor. | 2340 | 30TVL2104 | Rey Juan Carlos Database Sci-001-AG |
| Cen1 | POR | Central System. Serra da Estrela, Portugal. | 1900 | 29TPE1783 | Real Jardín Botánico de Madrid.CSIC. MA234833 |
| Mas | FR | Central Massif. Plomb du Cantal. Auvergne. Near the ski tracks. | 1560 | 31TDL8119 | Rey Juan Carlos Database Sci-001-RM |
| Pyr4 | FR | Pyrenees mountain range. Haut Conflent, Nohèdes, NE of Pico de la Pelada | 2190 | 31TDH3461 | Herbarium of Faculty of Farmacology, University of Barcelona. BCN64078 |
|  |  |  |  |  |  |
| Ari | GR | Aridaia mountain range. Mountain Tzena. | 2182 | 34TFL0142 | Herbarium of University of Patra. UPA822 |
| Bal3 | GR | Balkan-Rhodope mountain system. Mountain Falakron. | 1800 | 35TKF5580 | Herbarium of University of Patra. UPA1045 |
| Bal4 | GR | Balkan-Rhodope mountain system. Mountain Falakron. | 1800 | 35TKF5307 | Herbarium of University of Patra. UPA1455 |
| Bal5 | GR | Balkan-Rhodope mountain system. Mountain Falakron. | 1800 | 35TKF5586 | Herbarium of University of Patra. UPA1451 |
| Bal6 | GR | Balkan-Rhodope mountain system. Mountain Falakron. | 2060 | 35TKF5632 | Rey Juan Carlos Database Sci-001-PT |
| Bal1 | BU | Balkan-Rhodope mountain system. Rila mountains. | 1900 | 34TGM0365 | Herbarium of University of Sofia. SO100492 |
| Bal2 | BU | Balkan-Rhodope mountain system. Pirin mountains. | 2600 | 34TGM0229 | Herbarium of University of Sofia. SO104467 |
| Din | MAC | Dinaric Alps. Karadžica Planina mountain. | 2480 | 34TEM2771 | Herbarium of University of Sofia. SO 100916 |
| Ape1 | IT | Apennines mountain range. Abruzzos mountains. P. N. Gran Sasso. Barisciano. Valley Cupa, margin of the route. | 1950 | 33TUH8528 | Rey Juan Carlos Database Sci-002-RM |
| Ape2 | IT | Apennines mountain range. Abruzzos, Barisciano, after Vallicella and Valley Cupa. Parco Nazionale de Gran Sasso e Monti della Laga. | 1366 | 33TUH7979 | Herbarium Appeninnicum. APP11869. |
| Ape3 | IT | Apennines mountain range. Abruzzo. Majella National Park. | 2000 | 33TVG2225 | Herbarium of Vaccari. Museum of Natural History. University of Florence. FI5477. |
