# Supplementary material for: A glacial survivor of the alpine Mediterranean region: phylogenetic and phylogeographic insights into Silene ciliata Pourr. (Caryophyllaceae)
Source: PeerJ. 2015 Aug 20;3:e1193. doi: 10.7717/peerj.1193 (PMC4548490; doi:10.7717/peerj.1193)
Supplement: Table S2 — Primers used in this study for the amplification of the polymorphic cpDNA regions in Silene ciliata. “Region” lists all the polymorphic cpDNA regions of genus Silene or Silenea tribe, while “Primer” and “Sequence” provide all the names of the selected primers and their complete nucleotide sequence. “Reference” and “Silene sp.” indicate the bibliographical record of the study and species, where each set of primers was tested for the first time, respectively. Finally, “Program” describes the conditions in which each Polymerase Chain Reaction was performed. [file peerj-03-1193-s006.docx]

| Region | Primer |  | Sequence | Reference | *Silene* sp. | Program |
| --- | --- | --- | --- | --- | --- | --- |
| *trnS-trnG intron* | *trnS(GCU)* | F | GCCGCTTTAGTCCACTCAGC | (Hamilton, 1999; Sloan et al., 2012) | *S. vulgaris &* *S. latifolia* | 95ºC 5min; 40 ciclos: 95ºC 30 sec, 52ºC 1 min, 72ºC 1 min; 72ºC 7min |
|  | *trnG(UCC)* | R | GAACGAATCACACTTTTACCAC | (Ingvarsson & Taylor, 2002) |  |  |
| *matK* | *Kim3F* | F | CGTACAGTACTTTTGTGTTTACGAG | (Sloan et al., 2009) | *S. ciliata* | 95ºC 3 min, 40 ciclos: 95ºC30 sec, 49ºC 30sec, 72ºC 1 min; 72ºC 10min |
|  | *Kim1R* | R | ACCCAGTCCATCTGGAAATCTTGGTTC |  |  |  |
| *rbcL* | *rbcL1F* | F | ATGTCACCACAAACAGAAAC | (pers. comm. García-Fernández A.) | *S. ciliata* | 94ªC 3 min; 38 ciclos: 94ªC 1min, 50ªC 50 sec, 72ªC 1 min; 72ªC 8 min) |
|  | *rbcL724r* | R | TCGCATGTACCTGCAGTAGC |  |  |  |
|  | *S1_For* | F | ATGTCACCACAAACAGAGACT |  |  | 95ºC 3 min, 33 cycles: 94ºC 30sec; 55ºC 30sec, 72ºC 1min, 72ºC 10 min |
|  | *SI_Rev* | R | AAATCAAGTCCACCRCG |  |  |  |
| *rps16* | *rpsF2a* | F | CTTGAAGGACATGATCTGTTGTGGA | (Oxelman, Lidén &  Berglund, 1997) | tribe *Sileneae* | 95ºC 2 min; 33 ciclos: 95ªC 30 sec, 57ºC 1 min, 72ªC 2 min; 72ªC 7 min |
|  | *rpsR2* | R | CGATAGACGGCTCATTGGGATA | (pers. comm. Giménez-Benavides L., Prieto-Benítez S.) | *S. ciliata* |  |
| *psbA-trnH* | *trnH(GUG)* | F | ACTGCCTTGATCCACTTGGC | (Hamilton, 1999) | *S. vulgaris & S. latifolia* | 95ºC 4min; 35 ciclos: 95º30sec, 53ºC 45sec, 72ºC 45sec; 72ºC 7 min |
|  | *psbA* | R | CGAAGCTCCATCTACAAATGG | (Ingvarsson &Taylor, 2002) |  |  |
| *trnL* | *trnc* | F | CGAAATCGGTAGACGCTACG | (Taberlet et al., 1991) | *S. vulgaris & S. latifolia* | 95ªC 2,5 min; 38 ciclos: 95ªC 1 min, 53ªC 45 sec, 72ªC 1 min; 72ª 7 min |
|  | *trnd* | R | GGGATAGAGGGACTTGAACC | (Ingvarsson, Ribstein &  Taylor, 2003) |  |  |
| *trnL-trnF spacer* | *trne* | F | GGTTCAAGTCCCTCTATCCC | (Taberlet et al., 1991) |  | 95ªC 2,5 min; 38 ciclos: 95ªC 1 min, 53ªC 45 sec, 72ªC 1 min; 72ª 7 min |
|  | *trnf* | R | ATTTGAACTGGTGACACGAG | (Cotrim, 2001) | *S. nutans* |  |
| *psb-psbL spacer* | *psbE-RF* | F | TATCGAATACTGGTAATAATATCAGC | (Popp et al., 2005) | 23 Silene species | 95ºC 5min; 40 ciclos: 95ºC 30 sec, 56ºC 1 min, 72ºC 1 min; 72ºC 10 min |
|  | *petL-R* | R | ATAAGTCGTATCTTGTTYAGACCTA |  |  |  |
| *rpL32-trnL* | *trnLUAG* | R | CTGCTTCCTAAGAGCAGCGT | (López-Vinyallonga et al., 2012) | *S. sennenni* | 95ºC 3min; 34 ciclos, 94ºC 40sec, 54ºC 40sec, 72ºC 1min; 72ºC 10 min |
|  | *rpL32* | F | CAGTTCCAAAAAAACGTACTTC |  |  |  |
| *rpl16* | *rpl16Fc* | F | CAGTCAAGATATGATATATTGTTC | (pers. comm. García-Fernández A.) | *S. ciliata* | 94ºC 4 min; 34 cycles (94ºC 1 min, 55ºC 1 min, 72ºC 2:30 min), 72ºC 7 min |
|  | *rpl16R* | R | CCCTTCATTCTTCCTCTATGTTG |  |  |  |
| *trnS-trnSfm* | *trnfm* | R | CATAACCTTGAGGTCACGGG | (Minder, Rothenbuehler &  Widmer, 2007) | *S. dioica* | Temp. Annealing 62 |
|  | *trnSUGA* | F | GAGAGAGAGGGATTCGAACC |  |  |  |
| *rps4* | *rps4R2* | F | CTGTNAGWCCRTAATGAAAACG | (Taberlet et al., 1991) | Complete genome | 94ºC 3 min; 35 cycles (94ºC 15 sec, 50ºC 30 sec, 72ºC 1min), 72ºC 7 min |
|  | *trnLb* | R | TCTACCGATTTCGCCATATC |  |  |  |
