# Supplementary material for: A glacial survivor of the alpine Mediterranean region: phylogenetic and phylogeographic insights into Silene ciliata Pourr. (Caryophyllaceae)
Source: PeerJ. 2015 Aug 20;3:e1193. doi: 10.7717/peerj.1193 (PMC4548490; doi:10.7717/peerj.1193)
Supplement: Table S3 — Outgroup species, cpDNA region and GenBank Accession Numbers of the chosen species used for the Silene phylogeny analyses. [file peerj-03-1193-s007.docx]

| Outgroup Species | Accession Numbers for cpDNA Regions  (*rbcL, rps16, trnL)* |
| --- | --- |
| *Silene acaulis* | KC484105.1; KF305923.1; EF674238.1 |
| *Silene latifolia* | HQ590271.1; Z83171.1; EF674234.1 |
| *Silene nutans* | JN892747.1; EF061361.1; DQ061971.1 |
| *Silene vulgaris* | JN892985.1; EF674192.1; EF139653.1 |
| *Silene otites* | KC171366.1; EF061393.1; KJ746427.1 |
| *Silene schafta* | EF418563.1; Z83194.2; EU221631.1 |
| *Silene paradoxa* | KF527887.1; EF674190.1; EF674240.1 |
| *Silene uniflora* | HM850355.1; Z83173.1; EU221620.1 |
