# Supplementary material for: A glacial survivor of the alpine Mediterranean region: phylogenetic and phylogeographic insights into Silene ciliata Pourr. (Caryophyllaceae)
Source: PeerJ. 2015 Aug 20;3:e1193. doi: 10.7717/peerj.1193 (PMC4548490; doi:10.7717/peerj.1193)
Supplement: Table S4 — Accession numbers that have been obtained after the submission of Silene ciliata sequences in GenBank. The acronyms are used all through this study. Each of the accession number columns refers to a specific polymorphic cpDNA region. [file peerj-03-1193-s008.docx]

| Acronyms | *rbcL* accession number | *rps16* accession number | *trnL* accession number |
| --- | --- | --- | --- |
| Can1 | KM200337 | KM273179 | KM273207 |
| Can2 | KM200338 | KM273180 | KM273208 |
| Can3 | KM200339 | KM273181 | KM273209 |
| Ibe1 | KM200340 | KM273182 | KM273210 |
| Ibe2 | KM200341 | KM273183 | KM273211 |
| Pyr1 | KM200342 | KM273184 | KM273212 |
| Pyr2 | KM200344 | KM273186 | KM273214 |
| Pyr3 | KM200345 | KM273187 | KM273215 |
| Pyr5 | KM200346 | KM273188 | KM273216 |
| Cen2 | KM200347 | KM273189 | KM273217 |
| Cen3 | KM200348 | KM273190 | KM273218 |
| Cen1 | KM200349 | KM273191 | KM273219 |
| Mas | KM200350 | KM273192 | KM273220 |
| Pyr4 | KM200351 | KM273193 | KM273221 |
| Ari | KM200352 | KM273194 | KM273222 |
| Bal3 | KM200353 | KM273195 | KM273223 |
| Bal4 | KM200354 | KM273196 | KM273224 |
| Bal5 | KM200355 | KM273197 | KM273225 |
| Bal6 | KM200356 | KM273198 | KM273226 |
| Bal1 | KM200357 | KM273199 | KM273227 |
| Bal2 | KM200359 | KM273201 | KM273229 |
| Din | KM200361 | KM273203 | KM273231 |
| Ape1 | KM200362 | KM273204 | KM273232 |
| Ape2 | KM200363 | KM273205 | KM273233 |
| Ape3 | KM200364 | KM273206 | KM273234 |
